# Microbial biogeography of the eastern Yucatán carbonate aquifer

**DOI:** 10.1128/aem.01682-23

**Published:** 2023-11-02

**Authors:** Magdalena R. Osburn, Matthew J. Selensky, Patricia A. Beddows, Andrew Jacobson, Karyn DeFranco, Gonzalo Merediz-Alonso

**Affiliations:** 1 Department of Earth and Planetary Sciences, Northwestern University, Evanston, Illinois, USA; 2 Amigos de Sian Ka'an, and Consejo de Cuenca de la Península de Yucatán, Cancún, Quintana Roo, Mexico; Colorado School of Mines, Golden, Colorado, USA

**Keywords:** microbial biogeography, network analysis, karst, aquifer, microbiome, Yucatán

## Abstract

Constraining the spatial distribution of microorganisms and their ecological interactions is crucial for informing biogeochemistry. To that end, we explore horizontal and vertical patterns of microbial biogeography in the eastern Yucatán carbonate aquifer by examining the relative abundance of microbial taxa *via* 16S rRNA gene sequencing. As one of the largest anchialine groundwater systems on Earth, the density-stratified Yucatán aquifer consists of a meteoric lens overlying saline groundwater. The myriad sinkholes (cenotes) of the eastern peninsula lead into a vast network of subsurface conduits. Several studies describe microbial communities within specific regions of the aquifer, yet fundamental questions remain regarding the ecology and distribution of biogeochemically relevant microbes. Our analysis demonstrates that this aquifer hosts a distinct microbiome from nearby seawater, with regionalism observed across cave systems and vertical water column zones. We apply novel software to construct taxonomic co-occurrence networks at different scales and categorize highly connected groups of taxa into potential niches. Our network analysis approach suggests that ubiquitous, metabolically flexible taxa such as the family *Comamonadaceae* act as ecological linchpins across several niches, often directly or indirectly co-occurring with taxa capable of anammox (e.g., *Gemmataceae*), methanotrophy (e.g., *Methyloparacoccus*), or organoheterotrophy. Furthermore, communities from a deep, pit-like cenote open to the surface show the strongest niche partitioning between water column zones, differing from those encountered throughout the mostly dark and oligotrophic aquifer system, including another deep pit cenote with no direct surface opening. Our results suggest that members of a core microbiome could modulate different biogeochemical regimes depending on location, acting as reservoirs of metabolic potential in disparate environments of this groundwater system.

**IMPORTANCE:**

The extensive Yucatán carbonate aquifer, located primarily in southeastern Mexico, is pockmarked by numerous sinkholes (cenotes) that lead to a complex web of underwater caves. The aquifer hosts a diverse yet understudied microbiome throughout its highly stratified water column, which is marked by a meteoric lens floating on intruding seawater owing to the coastal proximity and high permeability of the Yucatán carbonate platform. Here, we present a biogeographic survey of bacterial and archaeal communities from the eastern Yucatán aquifer. We apply a novel network analysis software that models ecological niche space from microbial taxonomic abundance data. Our analysis reveals that the aquifer community is composed of several distinct niches that follow broader regional and hydrological patterns. This work lays the groundwork for future investigations to characterize the biogeochemical potential of the entire aquifer with other systems biology approaches.

## INTRODUCTION

Lourens Baas-Becking famously stated, “Everything is everywhere, but, the environment selects,” (1) to frame the concept of microbial biogeography, the study of the spatiotemporal distribution of microorganisms (2). The advent of high-throughput DNA sequencing technologies has enabled microbial ecologists to directly survey microbial communities from a myriad of habitats to test this now-famous hypothesis, including but not limited to soils (3, 4), marine settings (5 – 7), and aquifer systems (8, 9). A major focus of the field of microbial ecology is the interplay between microbes and their immediate environments. Interface environments with steep geochemical gradients are particularly key areas in which these interactions can be observed and parsed.

Anchialine aquifers are those that contain subterranean connections to the ocean and are common features of coastal carbonate platforms. The anchialine carbonate aquifer permeating the Yucatán Peninsula is one of the largest in the world and is stratified with a meteoric, freshwater to brackish, lens floating on denser, marine-derived saline groundwater (10, 11). These distinct water masses display sub-stratifications in salinity, conductivity, temperature, pH, redox potential, ionic composition, as well as dissolved oxygen, organic carbon, and inorganic carbon abundances (10 – 13). The depth of the halocline, the mixing zone between these water layers, increases with distance from the coast, increasing from ~ 5 to 10 m below the water table in Caribbean-adjacent sites to greater than 100 m in the middle of the peninsula (10, 14).

The Caribbean side of the Yucatán carbonate aquifer contains more than 1,500 km of mapped submerged horizontal conduits (caves) with sinkholes (cenotes) every km or less providing open connection to the surface, with these formed primarily *via* cave roof collapse (15, 16). Most (~97%) of the groundwater volume in the Yucatán aquifer is held within the highly porous carbonate matrix, although the majority (>99%) of the flux occurs in the caves (17), potentially allowing for horizontal dispersal of microbial communities across the aquifer. The extreme density stratification significantly limits the vertical transfer of material, except in areas adjacent to rock debris (e.g., roof collapses), where turbulent mixing can transfer some saline groundwater into the base of the meteoric water lens (11, 14, 18).

In addition to horizontal caves, there are also some vertically extensive submerged “pits” in the eastern Yucatán aquifer (11, 19), although these pit cenotes are most common in the northwestern regions of the peninsula ringing the Chicxulub impact crater (10, 20). These deep voids can be open or closed to the surface. In the NW of the peninsula, they typically lack horizontal conduits at any depth. This limits the lateral and horizontal transfer of material even further than is observed in horizontal conduits, isolating such pit environments from the rest of the aquifer (15, 21). Several pit cenotes in the eastern Yucatán do intersect with actively flowing shallow depth conduits.

These density-stratified competing hydrological regimes position the eastern Yucatán carbonate aquifer as an ideal natural laboratory to explore biogeographic patterns of microbial community distribution in the shallow subsurface. However, due to the inherent difficulties of obtaining samples from submerged caves beyond the open water zone, previous surveys describing the biogeochemistry and microbial communities of the water column have tended to focus on those in the immediate vicinity of the cenotes or deep pits (13, 20, 22 – 29). This focus leaves a distinct spatial and ecological gap as conduits represent the vast majority of the actively flowing aquifer habitat, connecting disparate regions of the groundwater system (17, 30).

Surface-derived organic matter entering open cenotes can accumulate in the halocline (13) and can be detected in conduit sediments over 200 m downstream (31). Its decomposition can create oxygen-poor conditions in the meteoric water and halocline zones (12). By contrast, the less-studied conduits and other isolated portions of the aquifer are distinct and can be comparably oligotrophic (32). The heterogeneity of the environments found within the Yucatán carbonate aquifer is reflected in the complex spatial distributions of the diverse microbiota it harbors (23 – 26, 29). Small- and large-scale biogeographic patterns have been observed which are affected by factors such as water column zone, human influence (25, 26), distance from the coast (29), and proximity to intruding plant roots (23). Not only do cenotes from the eastern and northwestern regions of the Yucatán Peninsula harbor distinct microbial communities, but phylum-level differences were observed between meteoric, halocline, and saline groundwater, regardless of cenote (25). Intra-phylum microbial biogeography has also been observed throughout the Yucatán aquifer, with different groups of sulfur-cycling Campilobacterota predominating in different sites (24). Despite this foundational work, the distribution and abundance of these and other biogeochemically relevant microbes throughout other environments within the eastern Yucatán carbonate aquifer remains an open question.

To understand the biogeochemical potential of the entire aquifer, we must first map the potentially uneven spatial distribution of taxa capable of diverse metabolic functions. Given the vast network of low-nutrient conduits that connect generally more eutrophic cenote entrances to the aquifer system (30), we hypothesize that a “core” microbiome exists throughout the aquifer. We posit that the local abundance of members of such a core microbiome reflects site-specific environmental contexts, such as distance from the coast, fluid geochemistry, and position in the water column. We explore the biogeography of the Bacteria and Archaea that colonize the water column of the eastern Yucatán carbonate aquifer through analysis of the relative abundance of 16S rRNA taxonomic marker genes in duplicate from 78 sampling points representing 12 unique caves. To investigate the drivers of microbial distributions, we consider the environmental context of each community, including cave type (pit vs conduit), cave system, distance from the Caribbean coast, aqueous geochemistry, and position in the water column. We employ a correlational network analysis-based approach to explore the prevalence and abundance of key, biogeochemically relevant taxa throughout the chosen study sites. Our analysis emphasizes identifying members of a “core” microbiome common throughout these diverse aquifer habitats to inform future studies of the biogeochemical potential of the entire Yucatán carbonate aquifer and similar anchialine ecosystems.

## MATERIALS AND METHODS

### Field sites and sampling

**Fig 1:**
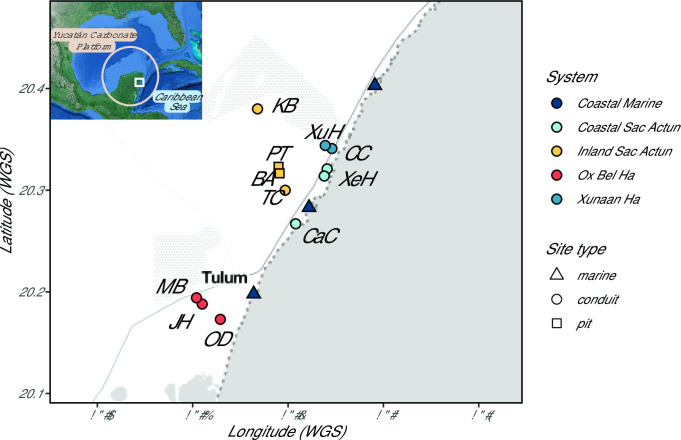
Study sites. Bacterial and archaeal communities from 66 water samples spanning the meteoric, halocline, and saline groundwater layers in the aquifer were analyzed in duplicate and compared to Caribbean seawater. Communities were sampled from 11 aquifer and 3 surface seawater sites near Tulum, Quintana Roo, Mexico. Sites are colored by cave systems according to previously mapped conduits. The meteoric water typically flows toward the coast, although decoupled saline groundwater may alternatively flow coastward and inland based on sea level (11). Refer to Table 1 in the main text for site descriptions and label IDs. Site Xel Ha (XeH) was sampled in two conduit branches. The base map was constructed using the R package ggmap (Kahle and Wickham, 2013).

In August 2019, a team of experienced cave divers led by P. Beddows obtained water samples (*n* = 78) from anchialine caves (*n* = 12) near the Caribbean coast in Quintana Roo, Mexico (Fig. 1; Table 1). This suite spans multiple cave systems including the Xunaan Ha system at the north end, inland and coastal portions of the Sac Actun system including a distinctive deep pit (The Pit), and the Ox Bel Ha system to the south. End-members and control samples include three surface seawater samples from ~10 m offshore and waist depth along the coast as well as a purified drinking water sample and a field control of that same water. Distance from the coast was measured for each cenote using the Google Earth Pro line segment tool.

**TABLE 1:** Site characteristics

System	Label	Site name	Sample site type	Entrance cenote distance inland (km)	Max. sampling depth (m)	Visible sulfide layer in halocline	Sequenced samples (# reps)	Unique water samples
Marine	SW	Seawater	Surface seawater	-	0.5	N	3 (6)	3
Xunaan Ha	CC	Chan Chemuyil	Closed conduit	0.8	15.2	N	4 (6)	6
	XuH	Xunaan Ha	Closed conduit	1.5	14.4	N	5 (7)	6
Coastal Sac Actun	CaC	Casa Cenote	Conduit, narrow coastal inlet	0.2	7.5	N	4 (7)	4
	XeH	Xel Ha - North	Conduit, wide coastal inlet	0.8	4.4	N	2 (3)	2
	XeH	Xel Ha - South	Conduit, wide coastal inlet	1	3.7	N	2 (4)	2
Inland Sac Actun	BA	Blue Abyss	Closed pit	4.7	60	N	8 (10)	10
	KB	K'oox Baal	Closed conduit	9.9	18.6	N	4 (7)	5
	TC	Tikim Chi	Closed conduit	3.1	19.2	N	6 (10)	8
	PT	The Pit	Open pit	5.7	60	N	12 (20)	13
Ox Bel Ha	JH	Jailhouse	Closed conduit	4.7	18.9	N	8 (14)	8
	MB	Maya Blue	Closed conduit	5.5	22	N	3 (4)	6
	OD	Odyssey	Closed conduit	2.2	15.5	N	6 (8)	8

Water samples were collected in ethanol-rinsed 1L glass autoclavable bottles by cave divers. Divers first partially filled the sampling bottles with cenote water to reduce buoyancy, then once at a given sampling depth, they opened, inverted, and voided the bottle with compressed air using a nozzle whip. Divers then swam upstream, rose in depth to expand the air and completely void the bottle, then inverted and filled the bottle with undisturbed upstream groundwater to minimize contamination. Sampling depth was recorded from wrist-mounted dive computers with a resolution of 0.1 m and set to EN13319 European CE standard for dive computers. The field control was collected during the dive to site Jailhouse (Fig. 1; Table 1), whereby an ethanol-rinsed sampling bottle filled with purified drinking water was taken along during the dive but unopened until filtering at the field laboratory. This sample was compared to a corresponding sample of purified drinking water that did not leave the laboratory and was filtered concurrently (discussed further in Section DNA Extraction. When feasible, the water column was characterized by multiparameter probes to measure depth, water temperature, pH, and conductivity. Two Hydrolab multiparameter probes were used including a Hydrolab MS5 and a Hydrotech OEM. These probes were calibrated daily for electrical conductivity (1.413 and 60 mS/cm) and pH (7, 10, and 4). The depths were set to freshwater settings and calibrated to 0 m depth with barometric pressure compensation each day.

Divers descended at a rate of 2 cm/s to allow for thermal equilibration of the probe. Samples were placed in a cooler on ice until transport to the field laboratory for filtering the same day.

### Water filtering

Prior to filtering and further analysis, the conductivity of an aliquot of unfiltered water was compared to field-based conductivity measurements to assess whether collection bottles were properly voided of surface water. Subsequently, ~1L of each water sample was filtered using a 0.2-µm Sterivex filter. Tubing was thoroughly pre-flushed with unfiltered samples to prevent cross-contamination. Filtered water for aqueous geochemical analysis (see Section Geochemical Analysis) was stored without headspace in the dark at 4°C until analysis. Filters were temporarily stored at −20°C in the field, transported on dry ice to the US in airline baggage, and at −80°C at Northwestern University prior to DNA extraction.

### Geochemical analysis

Geochemical measurements were made both in the field and back in the laboratory. Field measurement of total alkalinity was determined by Gran titration with standardized 0.02 N HCl on the 0.2-µm filtered samples within 1–2 days of collection. Daily titrations of a ~ 3,000 µeq/L Na_2_CO_3_ standard ensured measurement uncertainty remained below 5%. The pH probes were calibrated daily using pH 7, 4, and 10 solutions. Field measurements of conductivity were taken but repeated under laboratory conditions and compared to field values. Once back at Northwestern University, water samples were analyzed for major anions and cations at the Quantitative Bio-element Imaging Center using IC and inductively coupled plasma (ICP) emission spectroscopy methods. Anions (of which we report chloride and sulfate) were measured on a Thermo Scientific Dionex ICS-5000 + ion chromatograph and using a Dionex IonPac AS22 column. The analysis was run using an eluent of 4.5 mM sodium carbonate and 1.4 mM sodium bicarbonate and a Dionex AERS 500 Carbonate 4 mm electrolytically regenerated suppressor. Cations were measured *via* Thermo iCAP 7600 Inductively Coupled Plasma Optical Emission Spectroscopy. Analytical error on IC and ICP measurements is estimated at 5–10%. Conductivity was measured using a Myron Ultrameter II handheld meter calibrated within a range of 1.413 to 60.000 mS/cm both in the field and in the laboratory. All standards and samples were at an equivalent room temperature prior to the reported measurement.

### DNA extraction

DNA was extracted from filters using a previously described protocol (33). Thawed filters were split into two tubes and subsequently treated as duplicates for downstream analyses (Filter A and B in metadata tables). Split filters were then shredded into fine pieces using sterile, DNA-free scissors. To lyse cells, shredded filters were vortexed at high speed for 10 minutes in tubes containing sterile alumina beads and 2 mg mL^−1^ lysozyme buffer solution (25 mM Tris HCl, pH 8.0 and 2.5 mM ethylenediaminetetraacetic acid, pH 8.0 in DNA-free deionized water). To remove protein contaminates, the lysed cells were incubated at 55°C with 20 mg mL^−1^ proteinase K in TE buffer (Tris-HCl [10 mM] and EDTA [1 mM]) for 25 minutes. Samples were then incubated on ice for 15 minutes and were pelleted by centrifugation at 14,000 relative centrifugal force (RCF) for 10 minutes. A phenol:chloroform:isoamyl alcohol solution (25:24:1) was then thoroughly mixed with the supernatant. After centrifugation at 14,000 RCF for 10 minutes, the aqueous supernatant was then transferred to a sterile low-bind microcentrifuge tube. The supernatant was treated with ice-cold isopropyl alcohol (100%) and incubated at room temperature for 5 minutes to precipitate DNA, which was subsequently pelleted by centrifugation. The supernatant was then discarded, and the pellet was washed twice with ice-cold molecular-grade ethanol (70%) in between rounds of centrifugation. The DNA pellet was allowed to completely dry until it was resuspended in DNA-free deionized water and stored at −80°C until sequencing.

### DNA sequencing and quality control

Frozen DNA extracts were sent to the Environmental Sample Preparation and Sequencing Facility at Argonne National Laboratory (Lamont, IL). “Universal” bacterial and archaeal primers (515F/806R) were used to amplify the hypervariable V4 region of the 16S rRNA gene (34). Libraries were sequenced *via* Illumina MiSeq. The resultant 6,874,763 paired-end reads were imported into the QIIME2 (version 2020.6) software environment for processing (35). After demultiplexing using the *demux* command, a total of 6,260,931 reads passed the denoising and chimera check steps and were assigned into amplicon sequence variants (ASVs) with the DADA2 algorithm (36). Taxonomies were assigned to representative sequences using a pretrained Silva (v.138) classifier in QIIME2 (37 – 39). In addition to singleton taxa, contaminating ASVs were removed from the subsequent ASV-level taxonomic abundance table (40). The abundance of each ASV was normalized by the number of environmental samples. ASVs found in the negative controls from each DNA extraction batch were then identified in their corresponding samples. Such an ASV was considered a contaminant if its normalized abundance in each batch of samples was less than one order of magnitude than the normalized abundance in its corresponding negative control (40). A total of 54 contaminating ASVs, mainly Gammaproteobacteria, were identified and removed from the data set with this method. Following the removal of contaminants, we observed a total of 4,183 unique ASVs in our data set. The number of quality-controlled reads per sample ranged from 6,139 to 148,590 (Table S1). Based on the inflection point of an alpha rarefaction plot, we rarefied the ASV table to a sampling depth of 9,957 for downstream statistical analyses, with a total of 106 sequencing samples spanning 68 of the original 81 unique water samples (including seawater controls) included in the data set.

### Data analysis

Beta diversity was estimated at the ASV level by calculating Bray-Curtis dissimilarity on a matrix representing the relative abundance of ASV-level taxa (Table S4) and visualized in R by ordination *via* non-metric multidimensional scaling (NMDS) using the vegan package (41) and custom scripts. We estimated alpha diversity for each sample *via* the Shannon index (42) using a custom R script.

We applied “Biological Network Analysis and Learning” (“BNGAL”; https://github.com/mselensky/bngal), a custom R package and associated command-line tool to pre-process data and construct pairwise associative networks of ASVs with relevant metadata parameters. Networks are composed of “nodes” connected by “edges,” with graph theory examining the underlying substructures that may provide useful biological and/or ecological insight (43). There are many methods that can be used to construct networks from biological data (44), each with its own strengths and limitations (also recently summarized in Kishore et al. 2023). We constructed a network model of this data set that computes a correlation coefficient based on the co-occurrence of each possible pair of values, with the identity of a node corresponding to a single ASV (or environmental variable) and edges corresponding to the strength of its correlation coefficient with another node (e.g., the likelihood of the two to co-occur).

In this pipeline, the data are first prepared for network construction. Here, rarefied ASV counts of non-singleton taxa and environmental metadata for a given network are normalized to a common scale, relative abundance, or percentage of the total range for ASVs and metadata parameters, respectively. Then, the data are filtered using an “observational threshold” (*n*) such that *n* ≥ 5 for every pairwise relationship. In the physical context, this means that two ASVs must occur together at least five times to be included in the network, significantly reducing the inclusion of spurious correlations. A Spearman correlation matrix is then calculated using the rcorr command from the Hmisc R package (https://hbiostat.org/R/Hmisc), from which all possible network nodes (taxa or metadata parameter) and edges (connections) are identified. Edges are filtered to only include significant pairwise relationships with absolute Spearman correlation coefficients greater than 0.6 and *P*-values less than 0.05. To move beyond pairwise relationships and instead look at broader groups, we employ the statistical concept of “Edge betweenness” to further refine the grouping of ASVs and parameters within networks. Edge betweenness is defined as the number of shortest possible paths that pass through a given edge (45). Nodes that are highly connected will have low edge betweenness values, whereas those that are sparsely connected will have the highest values. Groups of nodes that are highly connected, termed “Edge betweenness clusters” (referred to in the text as ASV clusters for clarity), are computed by iteratively removing edges with the highest betweenness values until the network is divided into several densely intra-connected subnetwork clusters, revealing the internal structure of the entire network (45). The ASV clusters can be thought of as groups of ASVs and metadata parameters with a strong statistical likelihood of covariance, thereby approximating natural community structure. BNGAL employs the “igraph” software package (46) to build these networks. Networks are statically visualized with “igraph,” while interactive plots are generated with the “igraph”-based javascript library “visNetwork.”

## RESULTS AND DISCUSSION

We surveyed microbial communities present in 12 caves in the eastern Yucatán carbonate aquifer by 16S rRNA gene sequencing and compared them to three nearby surface seawater sites (Fig. 1; Table 1). We grouped caves based on known human-passable conduits as documented by cave divers and inferred flow paths as well as physical and chemical attributes into “cave systems” along the Caribbean coastline (Table 1). We acknowledge that only a fraction of these cave systems are currently mapped and as yet undocumented subsurface connectivity likely exists between adjacent systems. Due to the anchialine nature of the aquifer, cave samples are further classified using the measured conductivity into their water column zone of meteoric, halocline, or saline groundwater (Table S2). We define the halocline as portions of the water column where conductivity increases dramatically (typically by ~40 mS/cm) over a depth of 10 m or less, thereby separating groundwater with near-marine salinity from the overlying meteoric lens. Especially at sites within 1 km from the coast, the meteoric lens can be brackish (5–8 mS/cm; Fig. S1). From these sites, we identify 4,183 unique ASVs representing 82 total phyla, 202 classes, 538 orders, 917 families, and 1,953 genera. Of these, 145 ASVs were identified as Archaea from 12 unique phyla (Table S4).

### Microbial community compositions throughout the eastern Yucatán carbonate aquifer

**Fig 2:**
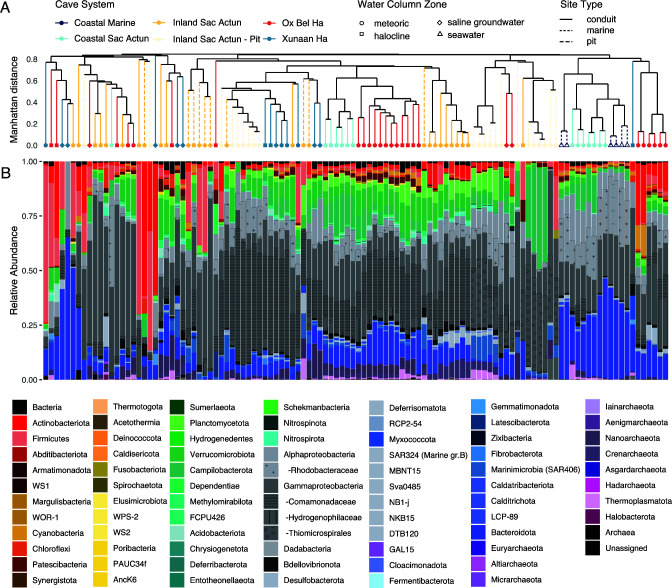
Microbial community composition of the eastern Yucatán carbonate aquifer. (**A**) “Complete” hierarchical cluster from a Manhattan distance matrix generated from an ASV-level taxonomic relative abundance table. Dendrogram ends and branches are colored by the Cave System (Fig. 1) and are shaped by the water column zone. Dendrogram branch line type corresponds to site type (**B**) Bacterial and archaeal community compositions, filled by phylum with key families and orders relevant to the discussion highlighted by pattern fill.

Across the data set, Proteobacteria (especially the classes Alpha- and Gammaproteobacteria) is the dominant phylum, representing 49.5% of sequences (Fig. 2B; Table S3). Other abundant phyla include Bacteroidota (11.4%), Verrucomicrobiota (5.6%), Firmicutes (5.4%), Actinobacteriota (4.7%), Campilobacterota (4.7%), Planctomycetota (2.9%), and Nanoarchaeota (2.1%), consistent with previous studies of subterranean conduits and estuaries (47). All other individual phyla represent less than 2% of the total observed reads each (Table S3). These numbers vary considerably by sample type and location. For instance, Verrucomicrobiota is relatively abundant in the aquifer microbiome regardless of cave system or water column zone (Table S3). By contrast, coastal seawater and heavily marine-influenced aquifer communities found in seawater controls and the coastal sites of the Sac Actun system are enriched in sequences of Alphaproteobacteria and Bacteroidota relative to other sites (Table S3). We also observe variable distributions of taxa within aquifer sites. Notably, halocline and saline groundwater communities from The Pit harbor significantly more of the SAR406 clade (Marinimicrobia) compared to the rest of the data set (Fig. 2B; Table S3).

To further explore how the microbial taxonomic landscape differs across sampling sites, we grouped communities at the ASV level *via* hierarchical clustering and then examined how those from different cave systems and water column zones cluster based on log_10_-transformed relative compositions (Fig. 2A). Our approach aims to deconvolve spatial trends and potential interactions between taxa, consistent with the recommendations set forth in a recent review of anchialine systems and submarine groundwater discharges (47). First-order branching divides microbial communities into two clusters, one whose shallower branches are highly dissimilar and a much larger one that exhibits clear regional patterns (Fig. 2). Most notably, marine-influenced communities from sites Casa Cenote and Xel Ha (Coastal Sac Actun; Table 1) cluster closely with surface seawater and is distinct from saline groundwater (Fig. 2). Communities from Caleta Xel Ha, a shallow and anchialine inlet on the Caribbean coast with north and south cave branches (Fig. 1), cluster much more closely with surface seawater communities than others in the aquifer (Fig. 2). Alongside surface seawater, communities from Xel Ha are marked by particularly high abundances of ASV mapped to the phyla Bacteroidota and Alphaproteobacteria compared to the rest of the aquifer (Fig. 2; marine mean 2.96 × 10^−3^ vs aquifer mean of 8.54 × 10^−3^; *P* = 1.12×10^−7^).

Intriguingly, none of the communities from Casa Cenote cluster with those from other Coastal Sac Actun sites despite broadly similar geochemical characteristics (Fig. S1; Table S2). Instead, they group primarily with halocline and meteoric communities from the Inland Sac Actun and Ox Bel Ha systems (Fig. 2). This pattern aligns with fundamental differences in the hydrology and geometry of the marine-influenced sites Casa Cenote and Xel Ha, which both circulate large volumes (~10^7^ m/year) of seawater (11, 14). However, because site Casa Cenote is partially roofed and has a comparatively narrower inlet, the tidal wedge of marine water extends only ~200 m from the coast there, compared to the 1–1.5 km distance in the wide and open Caleta site of Xel Ha (11, 14). Nevertheless, the relatively high compositions of the SAR_324 clade (Marine Group B) and Crenarchaeota, phyla commonly encountered in marine ecosystems (48, 49), distinguish Casa Cenote communities from others in the aquifer (Fig. 2), likely reflecting the influence of some intruding seawater.

By contrast, meteoric water communities across the data set tend to harbor relatively high proportions of Verrucomicrobiota, Nanoarchaeota, Planctomycetota, and Gammaproteobacteria (Fig. 2; Table S3). Meteoric water communities tend to be dominated by a single unclassified Gammaproteobacteria mapped to the family *Comamonadaceae*, which reaches 61.0% of the community at its most abundant (Table S4).

### Diversity

**Fig 3:**
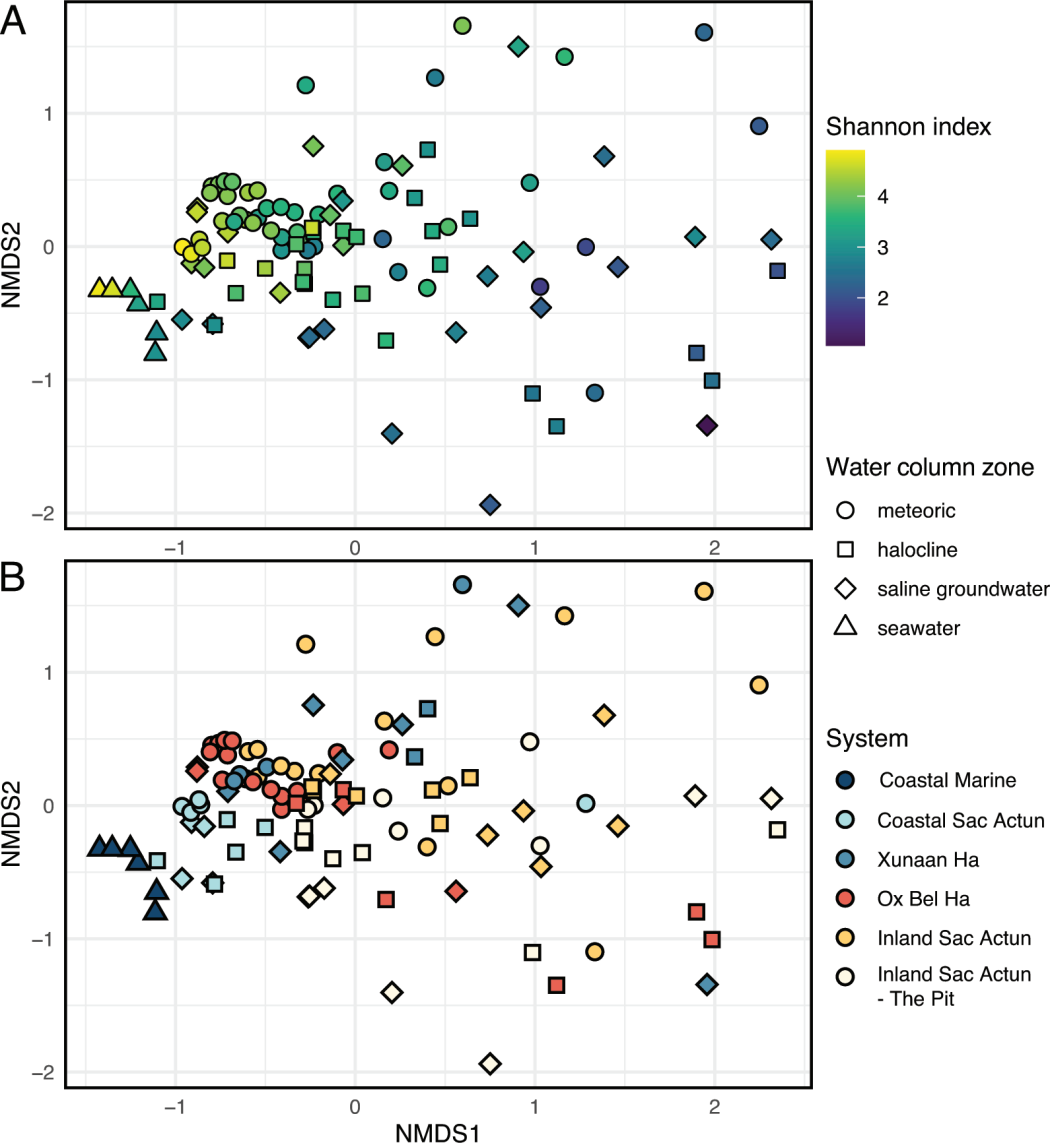
Patterns of diversity and regionalism displayed by microbial communities in the eastern Yucatán carbonate aquifer. NMDS analysis on a binary (presence/absence) matrix of bacterial and archaeal taxa binned at the ASV level (Table S4; solution stress = 0.189). (**A**) Communities with higher Shannon index values, indicating higher alpha diversity, tend to be more similar to each other than those with lower values. (**B**) Meteoric water aquifer communities tend to cluster regardless of the cave system (Fig. 1) while those from the halocline and saline groundwater exhibit more variability. Communities from seawater are distinct and can ordinate near those from Coastal Sac Actun.

We further investigated the alpha and beta diversities of communities in the eastern Yucatán carbonate aquifer and compared them to nearby seawater (Fig. 3). NMDS analysis of a presence-absence matrix at the ASV level (adapted from Table S4) shows grouping that corresponds to Shannon diversity as well as a cave system. Communities with the highest Shannon diversity in our data set are found grouped on the left and top of Fig. 3A with separation between seawater communities and those from caves. This suggests that groundwater microbial communities which are highly diverse, primarily from inland sites in Xunaan Ha, Inland Sac Actun, and Ox Bel Ha, are distinct from those found in seawater or the Coastal Sac Actun sites, agreeing with relative abundance-based hierarchical clustering results (Fig. 2). Furthermore, despite some variability, halocline and saline groundwater microbial communities from The Pit and other Inland Sac Actun sites cluster away from other regions in NMDS space coarsely divided by water column zone (Fig. 3B), suggesting distinct microbiomes.

Samples from Xunaan Ha, Inland Sac Actun, and Ox Bel Ha share similarities in that they are closed conduits sampled away from cenote openings. Since caves from these systems are geographically well distributed spanning ~25 km along the Caribbean coast (Fig. 1), we interpret the groupings observed in hierarchical clustering and NMDS results (Fig. 2A and 3B) as evidence of the presence of a diverse core microbiome in the eastern Yucatán carbonate aquifer.

The Pit, which is a 60-m-deep pit cenote open to the surface, contains a visible layer of hydrogen sulfide at the halocline that is within the photic zone (Table 1; Fig. S1) and has distinct microbial taxa in the halocline and saline groundwater (Fig. 2), with a marked increase in the abundance of sulfur-cycling microbes such as the SUP05 cluster of the Gammaproteobacteria (50) as well as the families *Sulfurovaceae* and *Sulfurospirillaceae* of the Campilobacterota (formerly classified under the Epsilonproteobacterota (51)) (Table S4). This diversity occurs despite hydrological inputs The Pit receives from the Inland Sac Actun conduits that intersect both inland and coastward sides of the site. We distinguish samples from The Pit from adjacent caves of Inland Sac Actun in figures for this reason. Notably, communities from another deep sulfidic pit structure in Sac Actun, the Blue Abyss, are distinct from The Pit and cluster separately (Fig. 2). A first-order environmental difference between The Pit and Blue Abyss is that the latter is close to the surface, preventing surficial organic matter and sunlight from directly reaching the water column. Strongly divergent microbial populations between similar and proximal pit cenotes may be common as was observed between two anchialine pits in the Bahamas (52).

Microbial community composition in the eastern Yucatán carbonate aquifer is influenced by both the water column zone and cave system (Fig. 2 and 3). Although aquifer communities tend to cluster by cave systems which are themselves subdivided by proximity to the coast, the water column zone also appears to drive some minor clustering within a given system (Fig. 2). Furthermore, a number of key taxa are shared among groundwater sites regardless of these variables. To delve deeper into these relationships and identify the taxa that comprise this putative “core microbiome” throughout the aquifer, we created a co-occurrence network model and examined the abundances of major co-occurring groups throughout the aquifer at different regional scales.

### A global co-occurrence network model of microbial niche space in the eastern Yucatán carbonate aquifer

**Fig 4:**
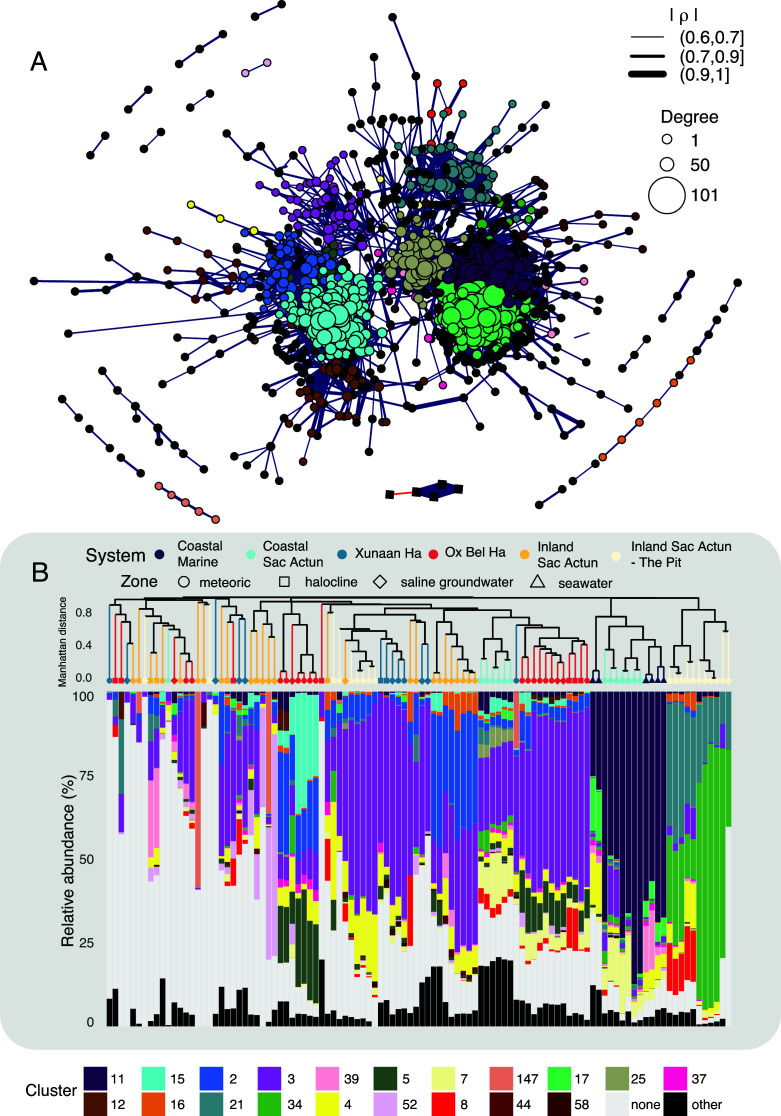
Global co-occurrence network of bacterial and archaeal ASVs from the eastern Yucatán carbonate aquifer. (**A**) Network colored by ASV clusters. Nodes are sized by degree (total number of co-occurrences) while the width of each edge corresponds to the strength of the Spearman correlation coefficient (ρ). Refer to Fig. S2 for an interactive version of this figure to probe individual relationships. (**B**) Relative abundance of ASV clusters. Samples are ordered *via* ASV-level hierarchical clustering.

We employ network analysis to model complex interactions within microbial communities, which has previously revealed ecologically relevant associations between groups of taxa (43, 53 – 56). Our network theory-based approach applied to the entire data set demonstrates the presence of several interconnected subnetworks of co-occurring taxa. Under our network model parameters, we observed 1,002 nodes (997 ASVs and 5 environmental variables) and 16,046 edges, representing 24% of unique ASVs (Table S4). These relationships are visualized in Fig. 4A where each node is scaled based on its degree of connectivity, and cooccurrence likelihood is shown by edge thickness. Highly connected clusters of nodes, as determined by Edge Betweenness Clustering (see methods), are color-coded and termed “ASV clusters” (Fig. 4A). The prevalence, abundance, and diversity of these ASV clusters varies by cave system and water column zone. We have visualized these variations across samples with hierarchical clustering and stacked bars illustrating the abundance of specific ASV clusters (Fig. 4B). We discuss the distribution and potential ecological significance of network node taxa from key ASV clusters in the following subsections.

#### Ubiquitous, universally abundant subnetworks in the eastern Yucatán carbonate aquifer

We interpret ASV clusters with high prevalence as those that harbor members of the “core microbiome” in the eastern Yucatán carbonate aquifer. Present in 98% of samples, ASV Cluster 3 is the subnetwork with the highest observed prevalence (Table S5). Intriguingly, the prevalence and abundance of ASV clusters show distinct patterns by cave system. For instance, the mean relative abundance of ASVs mapped to ASV Cluster 3 is 23.0% across all groundwater samples and only 0.86% in surface seawater samples (Fig. 4B; *P* < 2.2 × 10^−16^ *t*-test; Table S5). As such, we interpret ASV Cluster 3 to represent a component of the core microbiome in the aquifer. ASV Cluster 3 is comprised of 40 ASVs, primarily represented by uncultured members of the Nanoarchaeota (o. Woesearchaeales), Verrucomicrobiota (g. *Candidatus* Omnitrophicus), Campilobacterota (g. *Sulfurimonas*), and novel bacterial and archaeal ASVs only classifiable to the domain level (Table S7). Two ASV-level groupings of the phylum Methylomirabilota are also members of ASV Cluster 3 (Table S7), although they are only present in low abundance (<~1%) in the meteoric or upper halocline layers of most aquifer communities.

Uncultivated taxa compose some of the most abundant and ubiquitous taxa in the eastern Yucatán carbonate aquifer. For instance, an unclassified *Comamonadaceae* bin from ASV Cluster 3 is both the most prevalent (found in 91% of all samples, Fig. 2B) and most abundant bin in the Yucatán carbonate aquifer by median relative abundance (7.7%; Supplemental Table A). We examine median abundance when considering ubiquitous taxa to avoid the undue influence of high- or low-abundance outliers. The *Comamonadaceae* is a highly metabolically flexible family of Gammaproteobacteria commonly found in aquatic environments, with cultured representatives capable of aerobic heterotrophy, iron reduction, hydrogen oxidation, and denitrification (57). Despite its abundance, this *Comamonadaceae* bin only correlates with an uncultured marine taxon mapped to the NS9 marine group of Bacteroidota in the global network (Fig. 4A).

ASV Cluster 2 is similarly prevalent as ASV Cluster 3, with its taxa being present in 87% of communities, although it is noticeably less abundant in communities from marine-influenced sites and the Pit (Fig. 4). ASV Cluster 2 is a diverse subnetwork, harboring 49 ASVs from 13 distinct phyla (Table S7) and tends to be more abundant in meteoric water compared to other water column zones (Fig. 4B). A node classified to the hgcI clade of the Actinobacteriota family *Sporichthyaceae* exhibits the highest degree of co-occurrence in ASV Cluster 2, co-occurring with 32 taxa from several subclusters (Fig. 4A). Another taxon mapped to the same clade (hgcI_clade;uncultured_bacterium) co-occurs with 26 other nodes, although the only external ASV cluster connection it makes with another subcluster is with unclassified *Methyloparacoccus* (Fig. 4A). The high number of both internal and external ASV cluster connections exhibited by this clade of Actinobacteriota suggests that it is associated with multiple niches throughout meteoric water portions of the aquifer (45). Two Planctomycetota ASVs, classified as uncultured *Gemmataceae* and *Phycisphaeraceae* (CL500-3), are the most prevalent and abundant ASVs from ASV Cluster 2.

Taxa from ASV Cluster 2 often co-occur with those from ASV Cluster 15, a diverse and highly interconnected group of 118 taxa of mostly low abundance (< 1%), present in 64% of communities (Table S7). Within ASV Cluster 15, unclassified *Methyloparacoccus* (f. *Methylococcaceae*) is the taxonomic bin with highest prevalence (*n* = 24) with a median relative abundance of 0.075%, directly co-occurring with several taxa from ASV Cluster 2, including the two nodes representing members of the hgcI clade of *Sporichthyaceae* (Fig. 4A). ASV Cluster 15 is the most abundant in the meteoric water of Odyssey cave (Fig. 4B) and contains 118 taxa, represented by taxa capable of cycling C1 compounds such as *Methylococcaceae*, *Methylomonadaceae*, *Methylophilaceae*, and hydrogen oxidizers such as *Hydrogenophilaceae* strains. Sites with an abundance of ASV Cluster 2 taxa tend to also host others from ASV Cluster 4, which is present in 84% of samples (Table S5). The most prevalent taxonomic bin from ASV Cluster 4, unclassified *Rhodobacteraceae* (Table S7), only co-occurs with unclassified *Sphingomonadaceae* in the same subnetwork (ρ) = 0.68; Fig. 4A). The only other ASV in ASV Cluster 4, an ASV identified as *Candidatus planktoluna* (f. *Microbacteriaceae*), connects ASV Cluster 4 to ASV Cluster 2 through *Nitrosarchaeum*, *Sediminibacterium*, and unclassified *Gemmataceae* (ρ =0.65, 0.62, and 0.67, respectively).

Although the five taxa comprising ASV Cluster 7 tend to be encountered in low (≤1%) abundance, this sparsely interconnected subcluster is present in 73% of communities. Such taxa include an unclassified NS9 marine group taxon, *Candidatus Peribacteria*, and Campilobacterota (including two unclassified *Sulfurimonas* nodes and an uncultured *Arcobacteraceae*; Supplemental Table A). Of these taxa, the unclassified NS9 marine group and uncultured *Arcobacteraceae* are the most prevalent and abundant (Table S7). This subcluster is particularly abundant in Casa Cenote and Xel Ha (Fig. 4B).

#### Locally abundant subnetworks elucidate microbial biogeography

Some groups of “core microbiome” taxa exhibit high prevalence, being present in >50% of samples, but are only abundant in specific communities (Fig. 4B), suggesting that they proliferate only under appropriate environmental conditions. For example, taxa from ASV Cluster 21 and ASV Cluster 34 are present in 70% and 63% of communities, respectively (Table S7), but are by far the most abundant in halocline and saline groundwater communities from the deep and open site The Pit (Fig. 4B). ASV Cluster 21 contains 49 co-occurring taxa from 14 phyla, such as four bins from the SAR406 clade, Planctomycetota (especially OM190 and *Phycisphaeraceae*), and Gammaproteobacteria such as an uncultured bacterium bin of the SUP05 cluster (f. *Thioglobaceae*). By contrast, ASV Cluster 34 is less diverse, comprising nine nodes, of which five are Campilobacterota, represented by the families *Arcobacteraceae* (unclassified), *Sulfurimonadaceae* (g. *Thiovulum*), *Sulfurospirillaceae* (g. *Sulfurospirillum*), and *Sulfurovaceae* (g. *Sulfurovum*). Three other nodes in this subcluster are classified as Gammaproteobacteria, which represent uncultured *Ectothiorhodospiraceae*, the WHC3-3 group of *Nitrinocolaceae*, and *Thioglobaceae* (an unclassified SUP05 cluster bin; Supplemental Table A). Notably, ASV Cluster 21 acts to “bridge” the subclusters ASV Cluster 34 and ASV CLUSTER 11, which are most abundant in the saline groundwater of The Pit, and Xel Ha and seawater samples, respectively (Fig. 4). With 16 significant co-occurrences, the unclassified *Sulfurovum* bin is a particularly central node in ASV Cluster 21, exhibiting especially strong correlations with two members of the SAR406_clade from ASV Cluster 34 (ρ = 0.66 and 0.63), an unclassified *Spongiibacter* (ρ = 0.69), and a *Marinobacterium* metagenome from ASV Cluster 11 (ρ = 0.73; Fig. 4, Table S4). Notably, two ASVs from the SUP05 cluster, a genus of sulfur-oxidizing Gammaproteobacteria (50), are highly abundant in halocline and saline groundwater communities from The Pit. These ASVs are prevalent throughout the aquifer and seawater samples, but only reach above 5% of the community in The Pit, slightly below the halocline at the zone of sulfide accumulation (Fig. S1). One node classified to the SUP05 cluster is a part of ASV Cluster 21, while another node from the same group is a member of ASV Cluster 34 (Table S7).

Taxa from ASV Cluster 5 are similarly prevalent (in 64% of communities) but are instead most abundant in the meteoric water of Ox Bel Ha system (Fig. 4B). This subcluster contains 11 microbial nodes, with the most prevalent and abundant taxonomic bin representing unclassified *Hydrogenophilaceae*, a family mainly consisting of chemolithoautotrophs that are capable of various forms of sulfur oxidation as well as hydrogen oxidation (58). This node connects ASV Cluster 5 to the much more ubiquitous ASV Cluster 2 *via* the unclassified hgcI clade node of *Sporichthyaceae* (ρ = 0.63, Fig. 4A). ASV Cluster 5 taxa also tend to positively correlate with members of ASV Cluster 15, which is represented by taxa capable of cycling C1 compounds such as *Methylococcaceae*, *Methylomonadaceae*, *Methylophilaceae*, and hydrogen oxidizers including other *Hydrogenophilaceae* strains (Fig. 4A; Supplemental Table A).

Most co-occurring ASVs from seawater communities or marine-influenced coastal aquifer sites are neither abundant nor present in other parts of the aquifer (Fig. 4B; Supplemental Table A). This trend is illustrated by the distribution of taxa from ASV Cluster 11, a large subnetwork composing 81 ASVs that are mainly found in marine environments (Table S7). Marine Alphaproteobacteria such as the HIMB11 group of *Rhodobacteraceae* (59) or the SAR116 clade (60)*,* Bacteroidota such as *Cryomorphaceae* (61) or the photoheterotrophic NS5 marine group of *Flavobacteraceae* (62), and Gammaproteobacteria such as *Litoricolaceae* (63) only exceed 3% relative abundance in marine samples and are either absent or <0.5% in more inland aquifer communities, including the saline groundwater (Table S4). This implies that saline groundwater communities harbor a microbiome that is distinct from those in nearby coastal marine settings.

#### Cave system-level networks reveal site-specific co-occurrence patterns

While regional biogeography is present among co-occurring groups of microbes in the global network (Fig. 4B), environmental variables (depth, conductivity, and concentrations of sulfate, total sulfur, chloride, and alkalinity; Fig. 4A, squares) do not strongly correlate with any microbial nodes (Fig. 4A, circles). This is likely because significant variation in these environmental values between cave systems obscures potential pairwise trends with specific ASVs (Fig. S1). Distance from the coast did not correlate with any other nodes and is thus excluded from the global network. To probe more granular individual microbe-microbe and microbe-environmental variable co-occurrence relationships, we computed separate networks for each cave system (Fig. S3). In these more local subnetworks and subsequent subclusters, we find much stronger correlations to environmental variables and taxa correlations obscured in the global network. Key findings from this analysis are described below.

Coastal Sac Actun sites and seawater sites are evaluated together given their strong similarities in the global analysis (Fig. 2, 3B, and 4). These marine-influenced communities strongly cluster by site, with surface seawater and Xel Ha communities grouping together, apart from those within Casa Cenote (Fig. S4A). Here, microbial community composition (Fig. S4A) and individual co-occurrence patterns (Fig. S3A) are broadly consistent with variations in hydrological regimes. The wide, open Xel Ha channel allows for recirculating seawater to reach 1–1.5 km inland, while the bedrock topography and conduit morphology in Casa Cenote prevent the tidal seawater wedge from penetrating more than 200 m inland, resulting in the aqueous physicochemical characteristics of Casa Cenote to resemble the aquifer more closely (14). Unlike other sites, mangroves surround Casa Cenote, which could also affect the community composition at that site. Notably, the subcluster ASV Cluster S1_1 is abundant in the coastal aquifer sites (especially Casa Cenote) but is not in surface seawater (Fig. S4A). This subcluster contains many taxa prevalent throughout other areas of the aquifer, such as the uncultured *Comamonadaceae*, Campilobacterota such as *Arcobacteraceae* and *Sulfurimonas*, Omnitrophales, and others (Table S8). Alkalinity and water column depth have strong positive correlations with many members of ASV Cluster S1_1, consistent with the subcluster’s highest abundance in Casa Cenote and Xel Ha, which were sampled to greater depths than the surface seawater samples (Table 1). As terminal outflows of the meteoric water layer of the aquifer into the Caribbean, Casa Cenote, and Xel Ha exhibit higher alkalinity values than surface seawater, presumably due to the dissolution of carbonates from the rock matrix of the Yucatán platform (64).

Most aquifer communities exhibit more variable clustering patterns. Communities in the Xunaan Ha system, represented by the near-coast caves Chan Chemuyil and Xunaan Ha (Fig. 1), show some weak depth-dependent clustering, although they also tend to group by cave (Fig. S4B). The uncultured *Comamonadaceae* ASV only co-occurs with a highly central *Candidatus omnitrophicus* node in this region (Fig. S3B). By contrast, the Ox Bel Ha system comprises the sites Jailhouse, Maya Blue, and Odyssey (Fig. 1) and displays clustering primarily by site, although the water column zone also drives some clustering (Fig. S4C). Communities from Odyssey are well dispersed throughout the hierarchical cluster, although they tend to be most similar to the surface-most meteoric water of Jailhouse. Most Jailhouse communities contain high abundances (>39%) of ASV Cluster S3_5, which is dominated by the unclassified *Comamonadaceae* ASV (Table S8), a highly central node in this network (Fig. S4C). Here, this ASV shows a very strong positive correlation (ρ > 0.8) with six other ASVs, namely a *Hydrogenophaga* sp. (also within the *Comamonadaceae*), a *Sulfurimonas* sp., three mapped to the NS9 marine group, HdN1 of the *Halomonadaceae*, an uncultured *Cryomorphaceae*, and an *Arcobacteraceae* (Campilobacterota). Communities from the Inland Sac Actun System including sites Blue Abyss, K’oox Baal, and Tikim Chi (Fig. 1), cluster by cave and water column zone (Fig. S4D). Here, the unclassified *Comamonadaceae* bin is part of ASV Cluster S4_2, which is most abundant in meteoric water samples (Fig. S4D) and contains the unclassified *Comamonadaceae*, a taxon mapped to the OM190 group of the Planctomycetota, and two unclassified Woesearchaeles nodes (Table S8). Notably, the unclassified *Comamonadaceae* co-occurs with an unclassified *Sediminibacterium* bin (f. *Chitinophagaceae*; ρ = 0.65), which is a highly central node in the ASV Cluster S4_1 subnetwork (Fig. S3D).

Given the marked differences between The Pit and other inland Sac Actun sites, we evaluated co-occurrence trends within these samples separately. Compared to other aquifer communities, the co-occurrence network structure and ASV Cluster abundances here show strong niche partitioning by water column zone that is clearly distinct from other areas (Fig. S1 and S2). Microbial communities here are clearly distinguished by water column zone, with those in the meteoric lens clustering separately from halocline and saline groundwater communities (Fig. S4E). Here, the unclassified *Comamonadaceae* bin is a member of ASV Cluster S5_13 and is most abundant in the meteoric water lens, connecting 25 other nodes from multiple subclusters. It most strongly positively correlates with unclassified *Sediminibacterium* (ρ = 0.87) and the SH3-11 group of Verrucomicrobiota (ρ = 0.87). Total alkalinity positively correlates with the unclassified *Comamonadaceae* bin (ρ = 0.78; Fig. S3). All other environmental variables included in the network (total sulfur, sulfate, chloride, conductivity, and water column depth) negatively correlate with the unclassified *Comamonadaceae* node, reflecting its prevalence in the shallowest and most dilute meteoric water. Below the meteoric water, an unclassified bin related to the SUP05 cluster of S-cycling Gammaproteobacteria (50) is the sole member of ASV Cluster S5_20 and negatively correlates with the unclassified *Comamonadaceae* bin (ρ = −0.62), consistent with the highest abundance of this taxon in the halocline and saline groundwater of The Pit (Fig. S4E). This node exhibits relatively high degree (27) and positively correlates with other high-degree, single-node subclusters such as ASV Cluster S5_26 (another SUP05 cluster bin, ρ = 0.84), ASV Cluster S5_38 (uncultured *Sulfurospirillum*, ρ = 0.70), and ASV Cluster S5_7 (unclassified *Sulfurovum*, ρ = 0.76). The abundance of ASV CLUSTER 26_R5 is similar within the halocline and saline groundwater, while ASV Cluster S5_38 and ASV Cluster S5_7 are most abundant in the saline groundwater. Consistent with these spatial distributions, ASV Cluster S5_20 negatively correlates with alkalinity (ρ = −0.62) and positively correlates with conductivity, chloride, and total sulfur (ρ = 0.66, 0.64, 0.63, respectively).

### Microbiota in the eastern Yucatán carbonate aquifer inhabit distinct regional niches

Our analysis of ASV-level co-occurrence patterns across this data set provides clear evidence for the uneven distribution of microbial communities (Fig. 2) and co-occurrences (Fig. 4B) throughout the aquifer. Furthermore, co-occurrence patterns of key ASVs differ significantly between the global and local networks (Fig. 4B; Fig. S3), suggesting that members of the “core microbiome” are inherently flexible in their co-occurrence partners. This could be due to either the same taxon being involved in different biogeochemical processes across the aquifer or different partners filling the same niche space in different environments.

For instance, members of a metabolically flexible family of Gammaproteobacteria, *Comamonadaceae*, are some of the most ubiquitous and abundant taxa in the Yucatán carbonate aquifer (Fig. 2B), although which taxa they co-occur with varies by cave system. A single unclassified member of this family dominates meteoric water communities and is a member of ASV Cluster 3 in the global network (Fig. 4). Here, this ASV only co-occurs with an unclassified member of the NS9 marine group of Bacteroidota. This lack of network edge connections implies that this node does not represent a “keystone species” (45) in the aquifer despite its abundance and ubiquity. However, our local scale network analysis demonstrates instead that this node co-occurs with several different groups of microbes and exhibits distinct network node properties depending on the cave system (Fig. S3).

Members of the *Comamonadaceae* have been previously recognized as abundant “keystone species” in the surface-most portions of a schist aquifer (65). In that setting, *Comamonadaceae* ASVs positively correlated with nitrate concentrations, although their role in heterotrophic denitrification was determined to be unlikely due to limited organic carbon in the groundwater system (65). Genomic evidence suggests that many members of the *Comamonadaceae* are also capable of thiosulfate oxidation (66). In the Yucatán carbonate aquifer, ASVs from this family tend to be most abundant in meteoric water and positively correlate with taxa thought to be capable of anammox, hydrogen oxidation, and methylotrophy, although the specific partners vary by system (Fig. S3 and S5).

To illustrate, the unclassified *Comamonadaceae* bin positively correlates with taxa capable of anammox (namely Planctomycetota such as *Phycisphaeraceae, Gemmataceae*, and the OM190 clade) in every local network other than Xunaan Ha (Fig. S5). A taxon highly related to a metagenome representing the NS9 marine group of Flavobacteriales also co-occurs with unclassified *Comamonadaceae* in Coastal Sac Actun, Ox Bel Ha, and The Pit, which is also the only significant relationship displayed by the unclassified *Comamonadaceae* bin in the global network (Fig. 4B; Fig. S5). In the same regional networks, the unclassified *Comamonadaceae* bin also correlates with *Hydrogenophaga*, a genus of hydrogen oxidizers in the same family. Interestingly, the *Comamonadaceae* bin only correlates with methylotrophic bacteria (*Methylophilaceae*) in Coastal Sac Actun and The Pit (Fig. S5). In Xunaan Ha, the unclassified *Comamonadaceae* bin only correlates with a taxon classified as *Candidatus omnitrophus* (Fig. S5), an uncultured lineage that is common in other subsurface environments whose members are capable of various sulfur and nitrogen redox metabolisms (67, 68).

Based on its varied set of co-occurrences with taxa capable of several different functions (Fig. S5), we speculate that this unclassified *Comamonadaceae* bin acts as a reservoir of metabolic potential in the Yucatan carbonate aquifer. Nitrate reduction to nitrite is a commonly reported metabolism of cultured representatives of the *Comamonadaceae* (57). We therefore speculate that members of this abundant family could supply nitrite for anammox metabolisms fueled by ammonia produced *via* organic matter remineralization in the water column, especially in sub-oxic regions or microenvironments. Intriguingly, heterotrophic members of the order Flavobacterales have been previously implicated in the creation of sub-oxic microenvironments from organic matter degradation in an exposed ice sheet containing sulfurous deposits (69). Considering its co-occurrence patterns with the unclassified *Comamonadaceae* bin (Fig. S5), members of the NS9 marine group could fill a similar ecological role in areas of the Yucatán aquifer containing sufficient organic matter.

The unclassified *Comamonadaceae* bin also co-occurs with an unclassified *Sediminibacterium* bin in networks from Coastal and Inland Sac Actun (including The Pit) (Fig. S3 and S5). Although methane metabolism has not been observed in *Sediminibacterium* spp. to date, members of this organoheterotrophic genus of Bacteroidota have been previously observed to associate with methane-oxidizing communities in engineered biofilms and are suggested to utilize organic substrates produced from methane oxidation (70, 71). In our study sites, the unclassified *Sediminibacterium* bin co-occurs with the methylotrophic genus *Methylotenera* in Xunaan Ha and Inland Sac Actun (Fig. S3). Unlike the much more ubiquitous *Comamonadaceae* bin, the unclassified *Sediminibacterium* is absent in seawater and all saline groundwater communities except for one in Blue Abyss (Table S4), suggesting a distinctly terrestrial origin.

Where surface-derived organic carbon accumulates at the halocline, oxygen is rapidly consumed, and methane is produced by microbial methanogenesis (22, 72). This methane represents a major source of both energy and carbon in such areas. Isotopic mass balance from a previous survey estimates that 21% of the average cave shrimp diet in nearby Cenote Bang (1.8 km upstream of Maya Blue) is ultimately sourced from methanotrophic bacteria, even for those sampled in the caves downstream from the open Bang sinkhole (22). In sub-oxic microenvironments in the aquifer, methylotrophs and methanotrophs could produce hydrogen (73, 74) available for oxidation by *Comamonadaceae* spp. or others.

In the global network presented in this study, two nodes classified to the hgcI clade of Actinobacteriota co-occur with methylotrophic and methanotrophic bacteria in ASV Cluster 2 (Fig. 4A), which likely represents a distinct niche from ASV Cluster 3, the subcluster containing the unclassified *Comamonadaceae* (Table S7). Notably, both Actinobacteria link their subcluster, which contains two prevalent bins mapped to an uncultured *Gemmataceae* and *Phycisphaeraceae* (CL500-3) ASVs, to ASV Cluster 15 through an unclassified bin mapped to the genus *Methyloparacoccus* (Fig. 4A). *Methyloparacoccus* is a group of methanotrophic Gammaproteobacteria (75) while *Phycisphaeraceae* is associated with anammox in other settings (76). As such, the hgcI clade of Actinobacteriota may serve to bridge methane- and ammonia-cycling communities in the Yucatán carbonate aquifer.

Our results suggest that open pit cenotes with direct surface organic matter and sunlit haloclines host distinct communities with fundamentally different co-occurrence patterns than closed conduits and closed pits (e.g., Blue Abyss) in the Yucatán carbonate aquifer (Fig. 4; Fig. S3). We note that members of the SAR406 clade (Marinimicrobia), a group putatively capable of sulfur and nitrite oxidation (77, 78), are most abundant in the halocline of The Pit and are either absent or in low abundance elsewhere (Table S7). In the global network (Fig. 4A), several nodes belonging to the SAR406 clade co-occur with other putative sulfur oxidizers such as an unclassified bin of the SUP05 cluster, *Sulfurovum* (50, 79, 80) as well as an uncultured *Sulfurospirillum*, a genus whose cultured members exhibit a widespread capacity for elemental sulfur reduction and hydrogen oxidation (51, 81, 82). One cultured isolate of the SUP05 cluster reduces nitrate to nitrite coupled to thiosulfate oxidation (83), while *Sulfurovum* likely reduces nitrate coupled to sulfide oxidation in another setting in the Yucatán carbonate aquifer (24). These nodes are members of ASV Cluster 21 and ASV Cluster 34, which are most abundant in portions of The Pit at or below the halocline (Fig. 4A) where sulfur begins to accumulate (Fig. S1).

The unclassified SUP05 cluster bin is found in 49% of all communities (Table S7), including nearly all halocline and saline groundwater samples from every system, except for the site Maya Blue Cave in the Ox Bel Ha system. Intriguingly, this pattern is not as clear for other Campilobacterota, as the *Sulfurovum* and *Sulfurospirillum* bins are less prevalent (33 and 37%, respectively; Table S7). *Sulfurovum* is commonly encountered in highly sulfidic cave habitats (84, 85). Campilobacterota (specifically *Sulfurimonas* and *Sulfurovum*) have been previously observed to dominate Cenote Siete Bocas, a cenote ~15 km from the Caribbean coast that contains abundant surface organic matter, with sunlight penetrating the water column through several small (< 5 m) openings (24). In that site, geochemical and genetic evidence suggests *Sulfurovum* likely oxidizes sulfide *via* nitrate reduction (24, 79, 80, 86). In our global network, the unclassified *Sulfurovum* from ASV Cluster 34 positively correlates with an ASV closely related to an uncultured *Marinobacterium* metagenome (ρ = 0.73) and an unclassified *Spongiibacter* bin (ρ = 0.70) from ASV Cluster 11, thereby connecting the subclusters ASV Cluster 34 and ASV Cluster 11, which are most abundant in the saline groundwater of The Pit and marine-influenced sites, respectively (Fig. 4A). Notably, some cultured *Marinobacterium* spp. are capable of nitrate reduction as well (87), suggesting that communities in The Pit may follow a similar ecological paradigm.

Intriguingly, communities from Blue Abyss and The Pit are distinct (Fig. 2) although they are both vertically extensive pit cenotes in the Sac Actun system that contains accumulated sulfide and surface-derived organic matter, can exhibit oxygen-deficient haloclines and saline groundwater, and lie only ~900 m apart along the same inferred flow path (Fig. 1; Table 1). The major differences between these sites are the lack of both sunlight and the direct input of surficial organic matter entering the Blue Abyss, which is completely closed to the surface (Table 1). Despite the lack of a direct connection to the surface, some cave divers report the presence of bats in an air bell above the water of the Blue Abyss. Bat guano could provide a distinct source of organic matter that could affect microbial community composition in Blue Abyss, although the quantity of guano entering the aquifer in this site is unconstrained. Nevertheless, the surface-most meteoric water community in Blue Abyss (6.2 m depth) is unusually dominated by Actinobacteriota, especially the genus *Kocuria* (Table S4), where the relative abundance of this phylum reaches 62% (Table S3). Although Actinobacteriota are commonly found in soils, they are also major components of the bat guano microbiome (88, 89). Bat guano input could be an unaccounted lever on the microbial community composition of Blue Abyss that requires further investigation. Similar differences in microbial diversity of two blue hole sites in the Bahamas were observed previously (52), although specific taxa found in these sites are very different in that study being dominated by the phylum Chlorobi, suggesting that relatively subtle changes in geometry, hydrology, and light intensity of pit cenotes may have strong ecological impacts.

Most putative sulfur-cycling microbes in The Pit, namely the SUP05 cluster, *Sulfurovum*, and *Sulfurospirillum*, are either absent or in low abundance in Blue Abyss (Table S4). Instead, sub-halocline communities in Blue Abyss tend to be dominated by various members of the phylum Firmicutes, as well as *Acinetobacter venetianus* and *Pseudomonas pachastrellae* (Table S4). The latter two members of the Gammaproteobacteria order Pseudomonadales are both commonly found in marine settings and are capable of aerobic degradation of “recalcitrant” organic matter such as *n*-alkanes or other lipids (90 – 92). In the global network, these are the sole members of ASV Cluster 52, which does not correlate with any nodes in the larger network (Fig. 4A). As such, we speculate that *A. venetianus* and *P. pachastrellae* live within a restricted niche (45) reliant on the consumption of recalcitrant organic matter. The hydrology of the Yucatán aquifer is marked by active saline groundwater circulation, with the shallowest saline water shuttling back and forth. Although inflows from warm Caribbean surface water thermally equilibrate approximately 10 km inland (11), deeper saline groundwater flows are significant and likely continuous inland (14). We posit that the residence time of the saline water is long enough that the degradation of organic matter trapped at the density interface could lead to progressive anoxia with distance inland, especially in inland sites with less overall horizontal saline flow such as Blue Abyss (11, 19). In addition, since > 99% of groundwater flow in the Yucatán aquifer occurs in the conduits (17), we speculate that these hydrological factors contribute to a lack of widely available oxidants such as oxygen and nitrate in Blue Abyss to fuel the oxidation of sulfur compounds by populations of *Sulfurovum* and/or the SUP05 cluster (24, 83). This would correspond to the abundance of mostly anaerobic, fermentative Firmicutes in the saline groundwater of this site (Fig. 2; Table S4), none of which show significant co-occurrence patterns with other taxa and are thus excluded from the global and Inland Sac Actun networks (Fig. 4A; Fig. S3D).

Despite the presence of sunlight, sulfide, and organic matter, The Pit lacks well-described anoxygenic photosynthesizing taxa in its halocline and saline groundwater. Nonetheless, an unclassified bin of *Rhodobacteraceae*, a diverse family of Alphaproteobacteria often implicated in the sulfur cycle (59), is present in 83% of samples (Table S7) and is most abundant (~19%) in the meteoric water and halocline of The Pit, where sunlight is present (Fig. S6). Although we cannot assess its physiology with our methods, this family of Alphaproteobacteria is noted to host several genera of photosynthetic purple non-sulfur bacteria (59). This near-ubiquitous taxon also reaches almost 10% relative abundance in some communities near the dark halocline of Blue Abyss (Fig. S6). As such, this bin could represent a more flexible group of sulfur cycles present across many environmental conditions in the aquifer in a similar manner as the unclassified *Comamonadaceae* bin. *Sulfurovum*-dominated communities have been previously observed in surface glacial waters receiving sulfide from a cold seep despite the thermodynamic favorability of other redox metabolisms such as anoxygenic photosynthesis (69). There, intense aerobic heterotrophy is thought to establish sub-oxic microenvironments to allow *Sulfurovum* to oxidize sulfide with nitrate. In our sites that are in the flowing conduits (such as Jailhouse), suspended and dissolved organic matter can better disperse as it transits downstream, theoretically preventing the establishment of such conditions.

Our data suggest that differing hydrological regimes significantly affect the composition and interactions present in the sulfur-cycling communities in the Yucatán carbonate aquifer. Interestingly, the *Sulfurovum*, *Sulfurospirillum*, and SUP05 cluster nodes within ASV Cluster 21 and ASV Cluster 34 in the global network are only found in abundances higher than 1% in the halocline and saline groundwater of Odyssey (Table S4; Fig. S6). This conduit is directly downstream of Jailhouse; it is possible that smaller, mobile sub-oxic microenvironments created from the degradation of surface detritus that move downstream can support much lower abundances of the same sulfur cycles as those found in The Pit, eventually dissipating. However, Odyssey cave contains much higher abundances of ASV Cluster 15 in the global network (Fig. 4), which contains many methanogenic taxa such as *Methyloparacoccus* (93), suggesting that even non-pit cenotes could host fundamentally different biogeochemistries and community structures than their deeper counterparts.

### Conclusions

The Yucatán carbonate aquifer is one of the largest anchialine ecosystems on the planet, harboring a diverse microbiome that colonizes disparate groundwater habitats. Our analysis of the distribution of 16S rRNA genes in this well-connected aquifer demonstrates that regionalism exists across geographic distance, water column depth, and cenote/cave type, owning to the heterogeneous nature of the anchialine system. Significant compositional differences are noted between microbial communities in the saline groundwater and open seawater, despite the active circulation of Caribbean surface water ~10 km inland. Network analysis suggests that ubiquitous and metabolically flexible taxa such as *Comamonadaceae* act as “keystone species,” serving as reservoirs of metabolic potential throughout several potential niches. Furthermore, we observe biogeographic patterns in the distribution and co-occurrence patterns of key ASVs, in which communities within a given cave system tend to be more similar to each other than those from elsewhere. We also find that communities living in The Pit, a deep cenote that is open to the surface, are not representative of those encountered throughout the rest of the aquifer, whose habitat space is mostly constituted by dark, oligotrophic conduits. The Pit also demonstrates the strongest vertical stratification as well as niche partitioning between the meteoric, halocline, and saline groundwater layers among the chosen study sites. We conclude the Yucatán carbonate aquifer hosts a diverse and flexible core microbiome whose members proliferate under specific environmental or hydrological conditions. We speculate that this leads to the expression of distinct biogeochemical paradigms in different areas of the aquifer.

## Data Availability

Demultiplexed sequence data for all samples are freely available from the Sequence Read Archive (SRA) at the National Center for Biotechnology Information (NCBI). Data are referenced under BioSample accession numbers SAMN33725555-SAMN33725702 (BioProject PRJNA943420).

## SUPPLEMENTAL MATERIAL

Supplemental Figures and file index aem.01682-23-s0001.pdf This is a description of all supplemental files and includes supplemental figures and captions, except S2.

Figure S2 aem.01682-23-s0002.html Interactive global network (separate HTML file), filled by ASV cluster.

Table S1 aem.01682-23-s0003.csv Number of quality-controlled reads per sample.

Table S2 aem.01682-23-s0004.csv Sample metadata.

Table S3 aem.01682-23-s0005.csv Phylum-level abundance table.

Table S4 aem.01682-23-s0006.csv ASV-level taxonomic abundance data rarefied to a sampling depth of 9,957.

Table S5 aem.01682-23-s0007.csv Global network ASV cluster prevalence table.

Table S6 aem.01682-23-s0008.csv Global network ASV cluster per-sample abundance table.

Table S7 aem.01682-23-s0009.csv Global network ASV cluster memberships for each ASV-level taxon.

Table S8 aem.01682-23-s0010.xlsx List of taxa node ASV cluster membership for each cave system. Saved as an Excel table with each tab corresponding to one region.

ASM does not own the copyrights to Supplemental Material that may be linked to, or accessed through, an article. The authors have granted ASM a non-exclusive, world-wide license to publish the Supplemental Material files. Please contact the corresponding author directly for reuse.
