# Supplementary material for: Microbial biogeography of the eastern Yucatán carbonate aquifer
Source: Appl Environ Microbiol. 2023 Nov 2;89(11):e01682-23. doi: 10.1128/aem.01682-23 (PMC10701671; doi:10.1128/aem.01682-23)

Microbial Biogeography of the Eastern Yucatán Carbonate Aquifer
Supplemental Information

Supplemental Tables

All supplemental tables are available as separate comma-separated value (CSV) files in the provided YucatanMicroBiogeo_SupplementalInfo folder unless otherwise specified.

Table S1: Number of quality-controlled reads per sample.
Table S2: Sample metadata.
Table S3: Phylum-level abundance table.
Table S4: ASV-level taxonomic abundance data rarefied to a sampling depth of 9,957.
Table S5: Global network ASV cluster prevalence table.
Table S6: Global network ASV cluster per-sample abundance table.
Table S7: Global network ASV cluster memberships for each ASV-level taxon.
Table S8: List of taxa node ASV cluster membership for each cave system. Saved as an Excel table with each tab corresponding to one region.

Supplemental Figures

All supplemental figures are included in this document unless otherwise specified.

Figure S1: Geochemical variables across study sites.
Figure S2: Interactive global network (separate HTML file), filled by ASV cluster.
Figure S3: Regional co-occurrence networks, filled by ASV cluster.
Figure S4: Relative abundance of regional network ASV clusters.
Figure S5: Co-occurrences of the unclassified *Comamonadaceae* bin with network nodes across the global and regional networks.
Figure S6: Relative abundance of selected taxa putatively capable of sulfur cycling.

Figure S1: Geochemical variables across study sites. A. Conductivity profiles of each site reported in mS/cm. Points are shaped based on the water column zone and colored by Cave group.

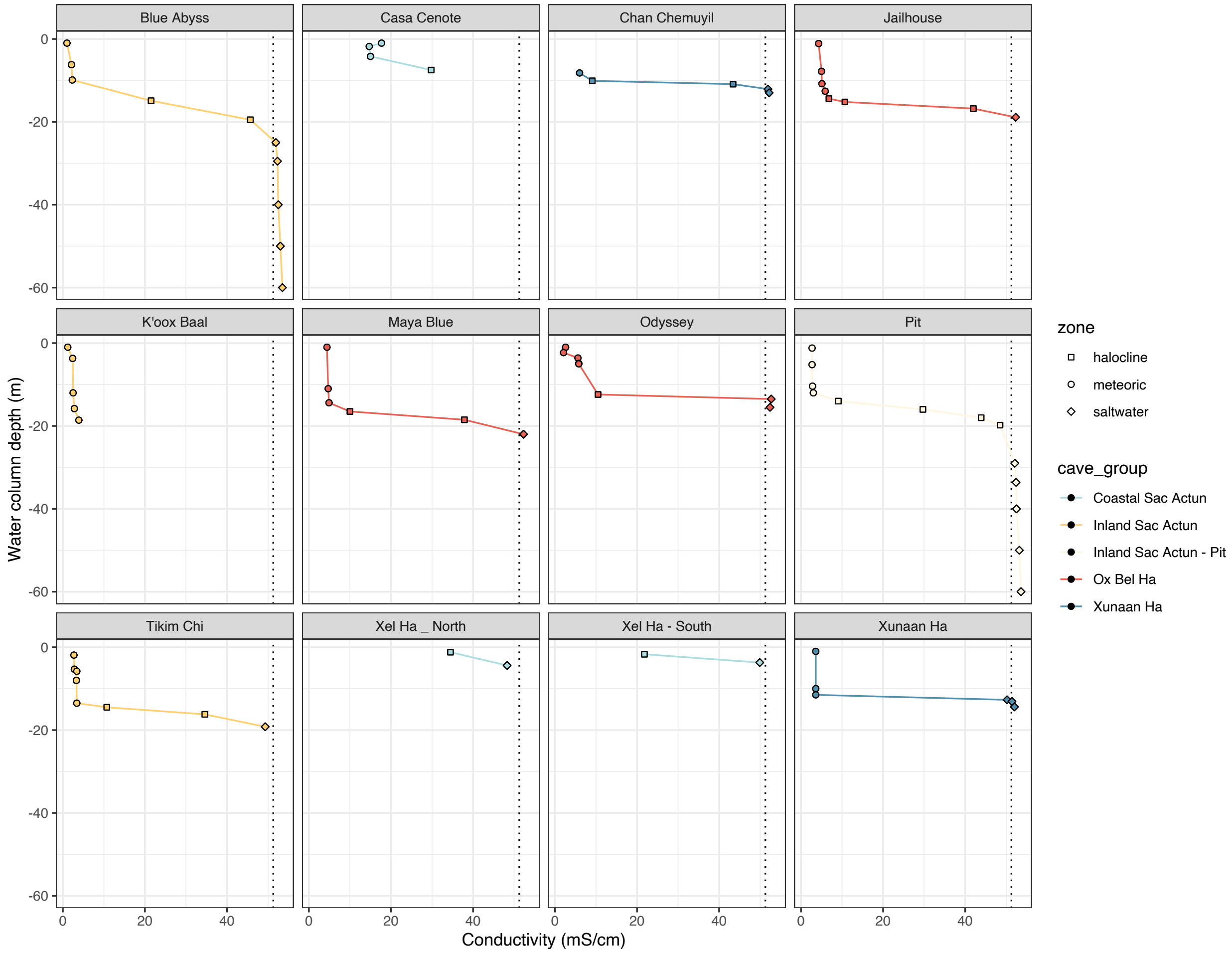

B: Ionic composition of water samples plotted by cave. Seawater concentrations measured in controls are show by dotted vertical lines. All values are reported in milliequivalents per liter (mEq/L), but sulfate is shown at 10X exaggeration for more direct comparison. Symbols reflext reported water column zone for each sample.

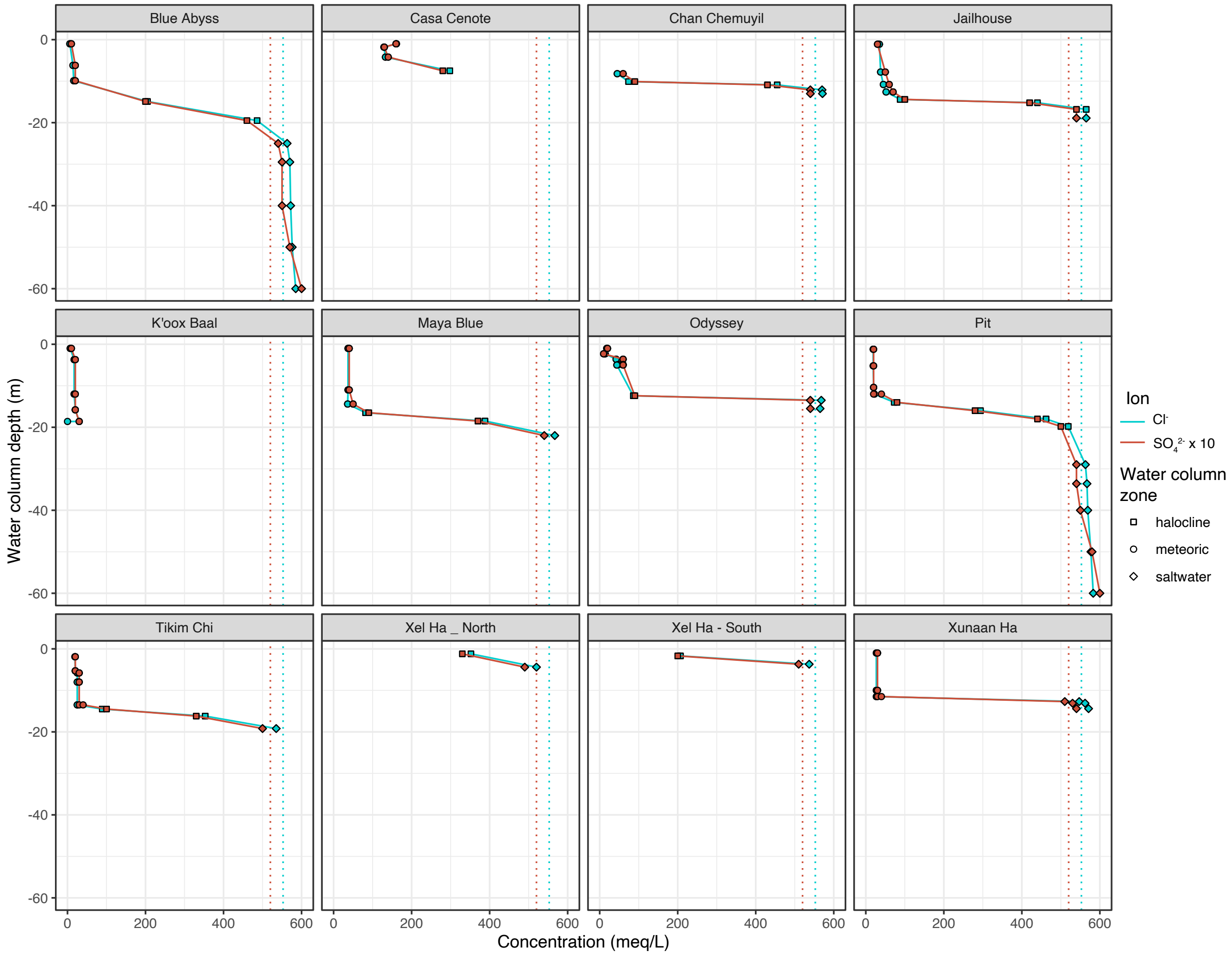

Figure S2: Interactive global network, filled by edge betweenness cluster (EBC). Open the attached file “SuppFig2.html” in a web browser to probe individual pairwise relationships. Refer to Figure 4B in the main text for a static version of this figure.

Figure S3: Regional co-occurrence networks. S3A: Region1. S3B: Region2. S3C: Region3. S3D: Region4. S3E: Region5. Refer to the main text for discussion.

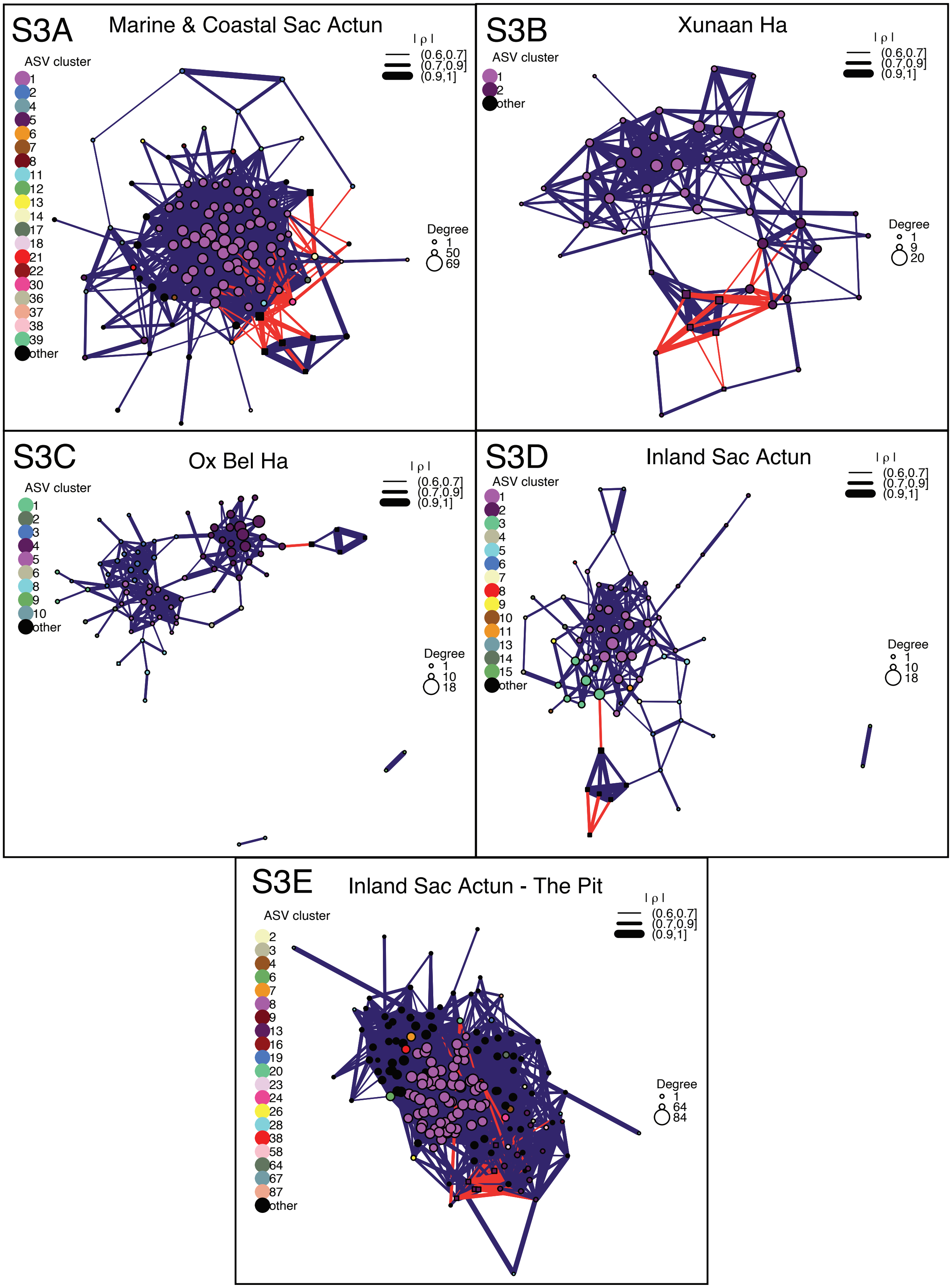

S3A
Marine & Coastal Sac Actun
ASV cluster
1
2
4
5
6
7
8
11
12
13
14
17
18
21
22
30
36
37
38
39
other
| ρ |
(0.6,0.7]
(0.7,0.9]
(0.9,1]
Degree
1
50
69
S3B
Xunaan Ha
ASV cluster
1
2
other
| ρ |
(0.6,0.7]
(0.7,0.9]
(0.9,1]
Degree
1
9
20
S3C
Ox Bel Ha
ASV cluster
1
2
3
4
5
6
8
9
10
other
| ρ |
(0.6,0.7]
(0.7,0.9]
(0.9,1]
Degree
1
10
18
S3D
Inland Sac Actun
ASV cluster
1
2
3
4
5
6
7
8
9
10
11
13
14
15
other
| ρ |
(0.6,0.7]
(0.7,0.9]
(0.9,1]
Degree
1
10
18
S3E
Inland Sac Actun - The Pit
ASV cluster
2
3
4
6
7
8
9
13
16
19
20
23
24
26
28
38
58
64
67
87
other
| ρ |
(0.6,0.7]
(0.7,0.9]
(0.9,1]
Degree
1
64
84

Figure S4: Relative abundance of regional network node ASV clusterss. Cave-specific codes and cave system groups are as follows:

S4A Marine and Coastal Sac Actun Systems: CaC = Casa Cenote, XeH = Xel Ha, SW = Seawater

S4B Xunaan Ha System: CC = Chan Chemuyil, XuH = Xunaan Ha

S4C Ox Bel Ha System: MB = Maya Blue, OD = Odyssey, JH = Jailhouse

S4D Inland Sac Actun System: KB = K'oox Baal, BA = Blue Abyss, TC = Tikim Chi

S4E Inland Sac Actun – The Pit samples: PT = The Pit

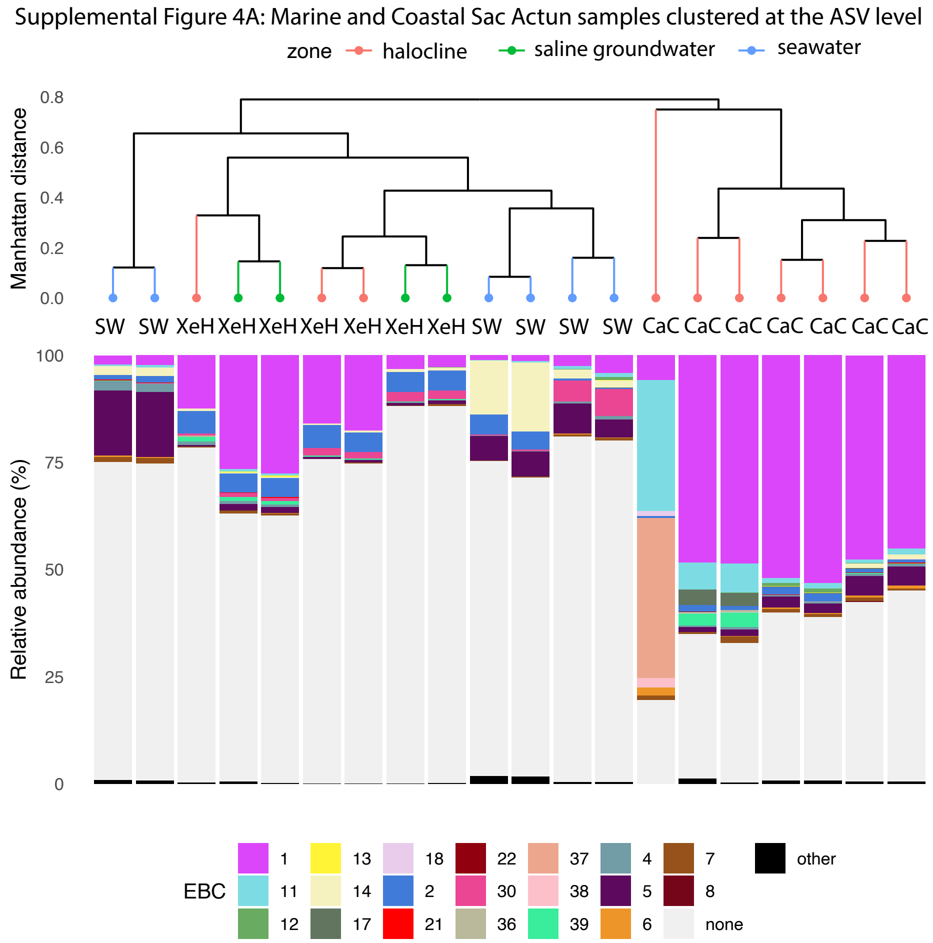

Supplemental Figure 4B: Xunaan Ha system samples clustered at the ASV level

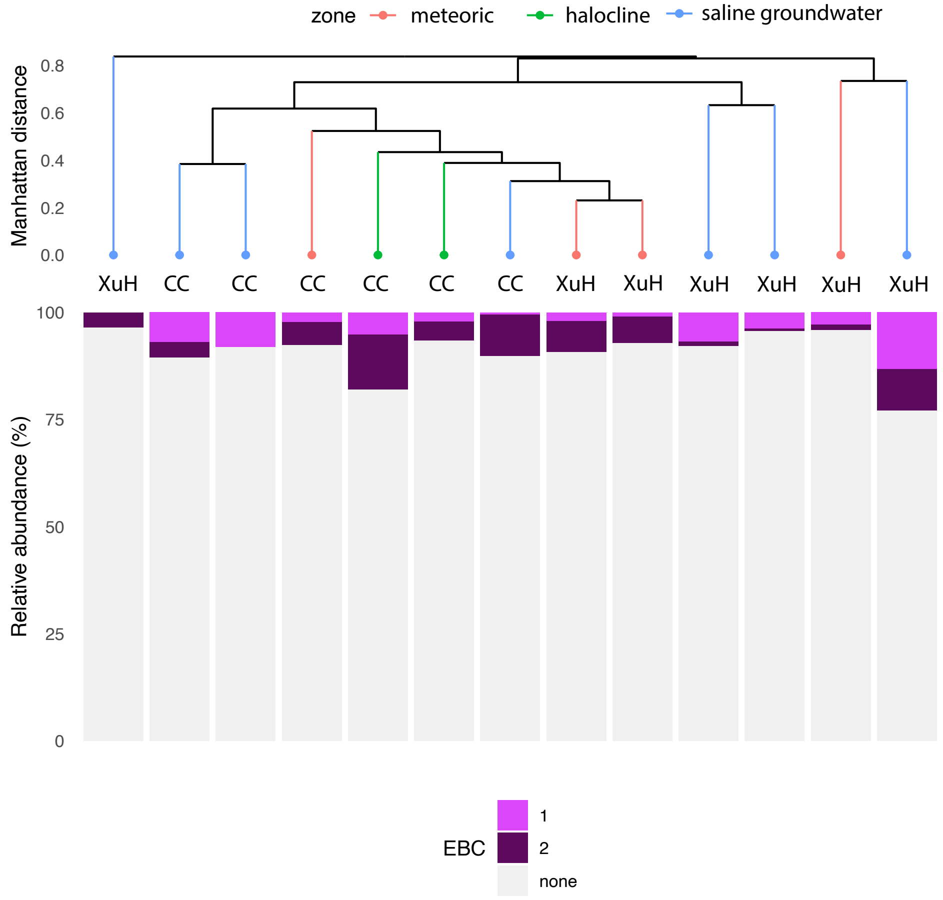

Supplemental Figure 4C: Ox Bel Ha system samples clustered at the ASV level

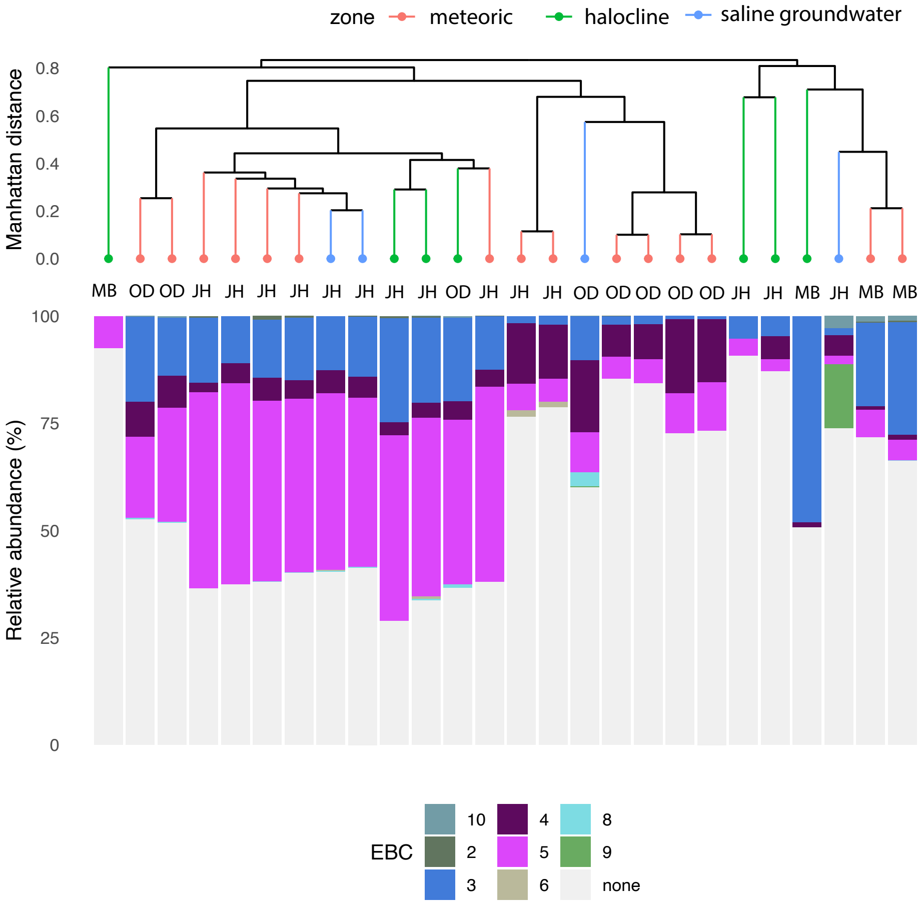

Supplemental Figure 4D: Inland Sac Actun system samples clustered at the ASV level

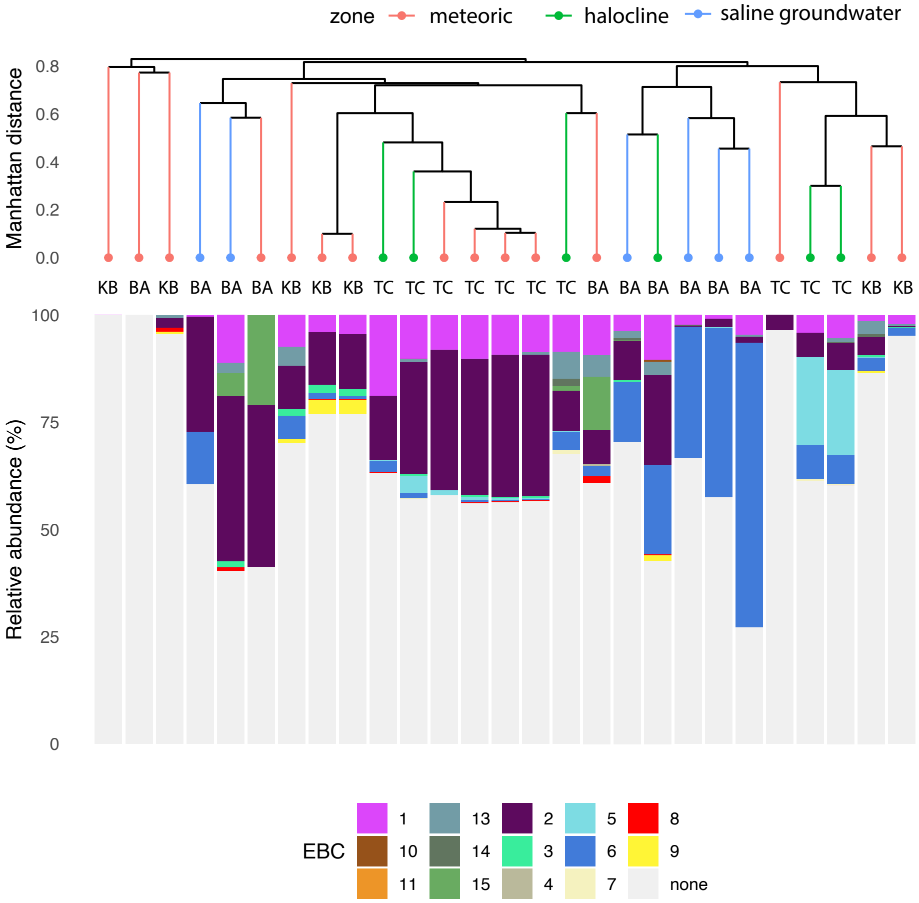

Supplemental Figure 4E: Inland Sac Actun - Pit system samples clustered at the ASV level

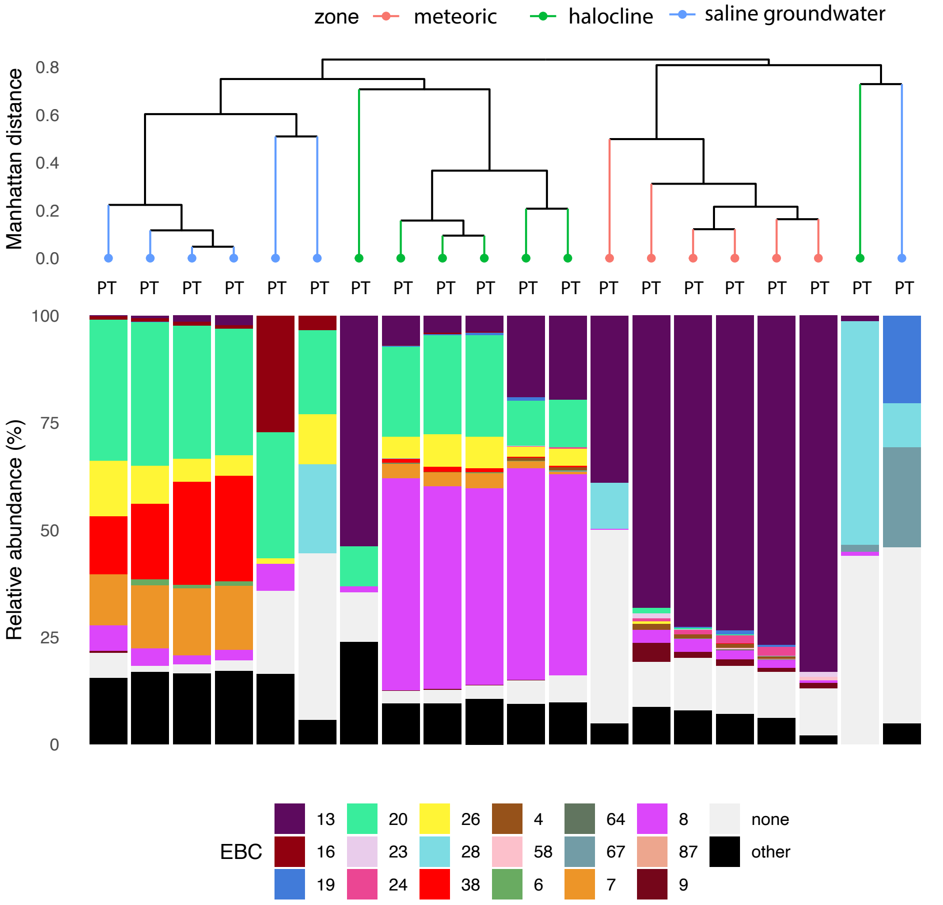

Figure S5: Co-occurrences of the unclassified *Comamonadaceae* bin with other network nodes across the global and regional networks.

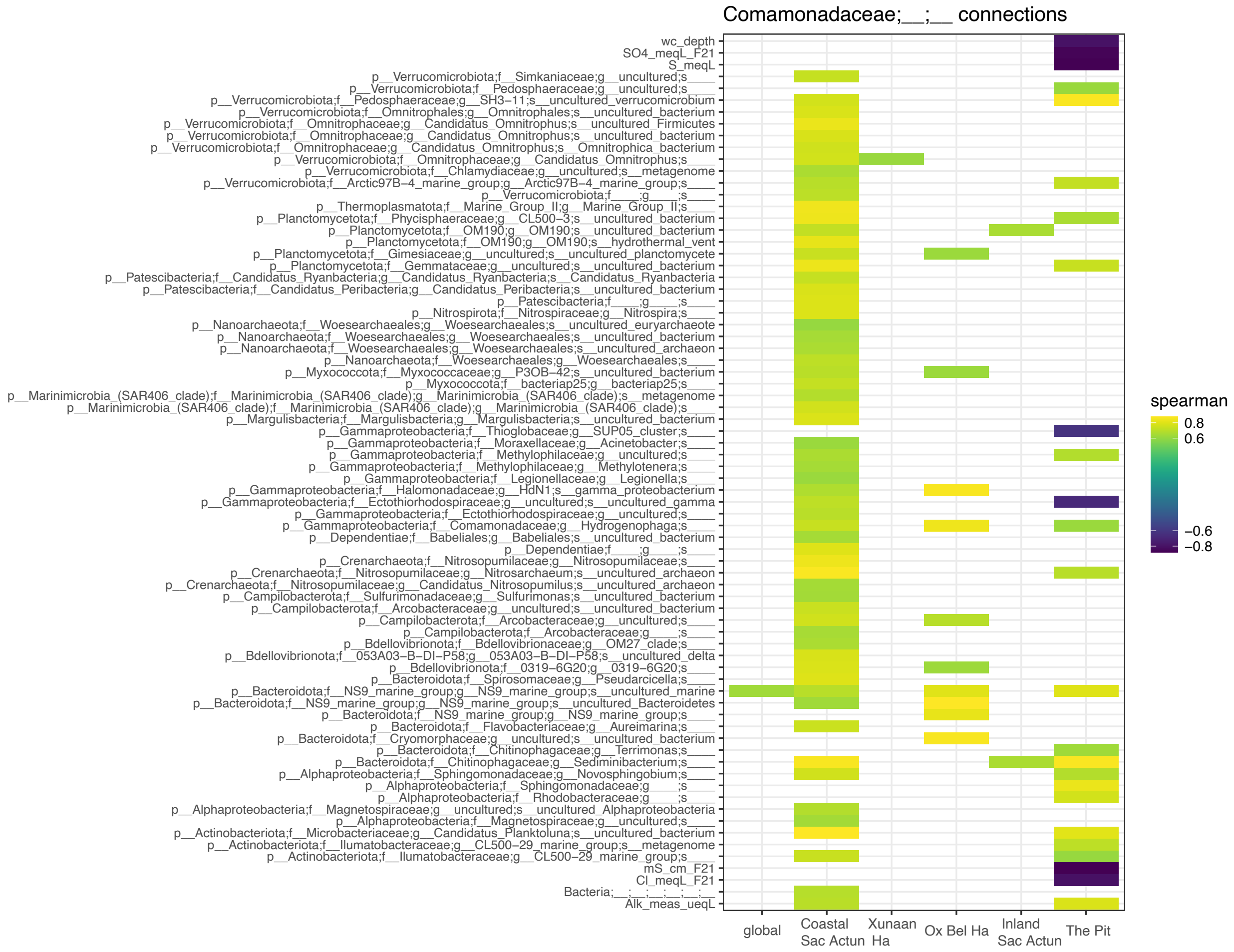

Figure S6: Relative abundance of selected taxa putatively capable of sulfur cycling. Conductivity is represented as a dimensionless dashed line to visualize density stratification.

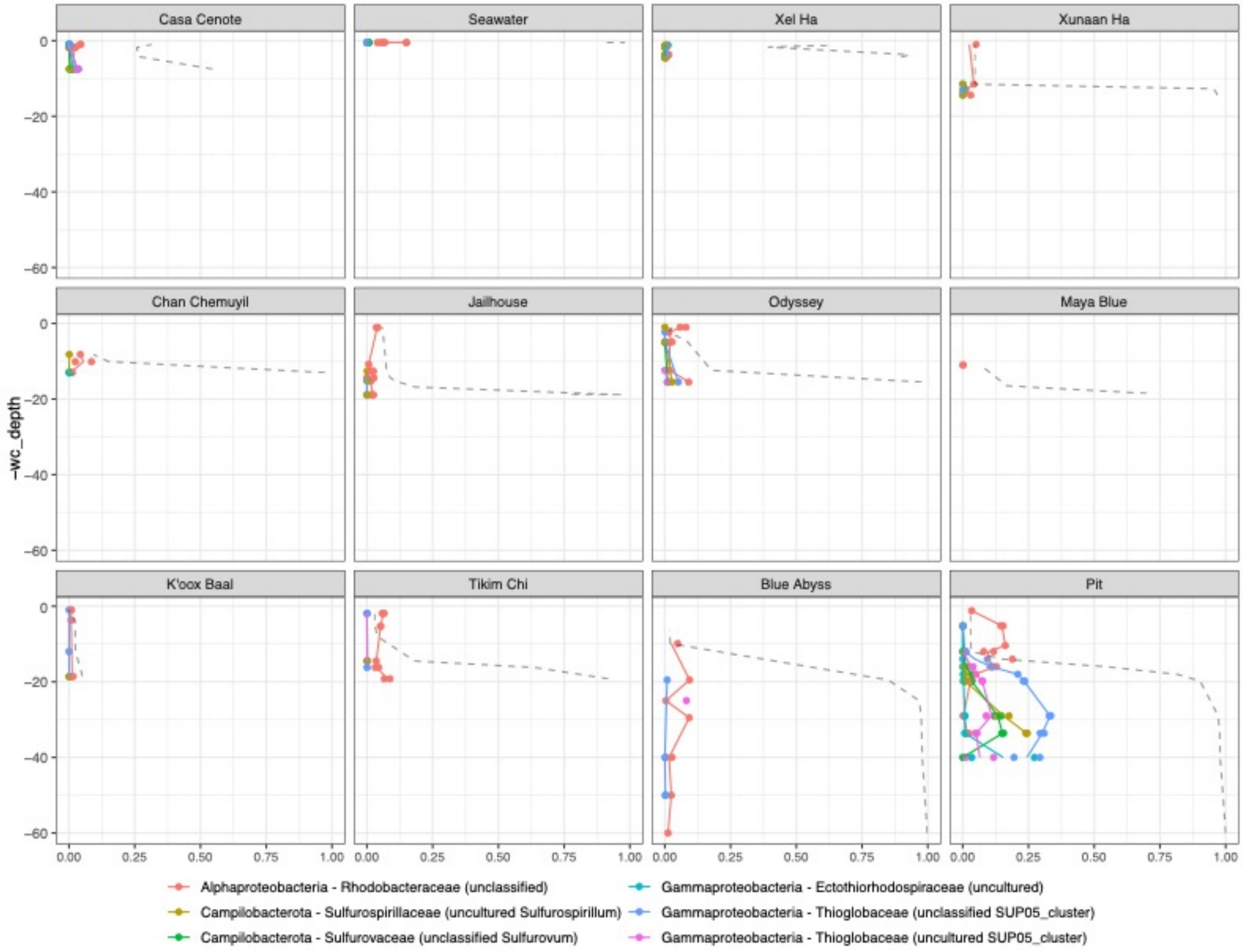

Supplement: Supplemental Figures and file index — This is a description of all supplemental files and includes supplemental figures and captions, except S2. [file aem.01682-23-s0001.pdf]
